# Adipocyte-derived lactate is a signalling metabolite that potentiates adipose macrophage inflammation via targeting PHD2

**DOI:** 10.1038/s41467-022-32871-3

**Published:** 2022-09-05

**Authors:** Tianshi Feng, Xuemei Zhao, Ping Gu, Wah Yang, Cunchuan Wang, Qingyu Guo, Qiaoyun Long, Qing Liu, Ying Cheng, Jin Li, Cynthia Kwan Yui Cheung, Donghai Wu, Xinyu Kong, Yong Xu, Dewei Ye, Shuang Hua, Kerry Loomes, Aimin Xu, Xiaoyan Hui

**Affiliations:** 1 State Key Laboratory of Pharmaceutical Biotechnology, The University of Hong Kong, Hong Kong, China; 2 Department of Medicine, The University of Hong Kong, Hong Kong, China; 3 School of Biomedical Sciences, The Chinese University of Hong Kong, Hong Kong, China; 4 Department of Endocrinology, Jinling Hospital, School of Medicine, Nanjing University, Nanjing, China; 5 The First Affiliated Hospital of Jinan University, Guangzhou, China; 6 Key Laboratory of Regenerative Biology, Guangdong Provincial Key Laboratory of Stem Cell and Regenerative Medicine, Guangzhou Institutes of Biomedicine and Health, Chinese Academy of Sciences, Guangzhou, China; 7 China-New Zealand Joint Laboratory on Biomedicine and Health, Guangzhou, China; 8 Guangdong Pharmaceutical University, Guangzhou, China; 9 School of Biological Sciences and Maurice Wilkins Centre, University of Auckland, Auckland, New Zealand; 10 Department of Pharmacy and Pharmacology, The University of Hong Kong, Hong Kong, China

**Keywords:** Metabolic syndrome, Chronic inflammation, Monocytes and macrophages, Interleukins

## Abstract

Adipose tissue macrophage (ATM) inflammation is involved with meta-inflammation and pathology of metabolic complications. Here we report that in adipocytes, elevated lactate production, previously regarded as the waste product of glycolysis, serves as a danger signal to promote ATM polarization to an inflammatory state in the context of obesity. Adipocyte-selective deletion of lactate dehydrogenase A (*Ldha*), the enzyme converting pyruvate to lactate, protects mice from obesity-associated glucose intolerance and insulin resistance, accompanied by a lower percentage of inflammatory ATM and reduced production of pro-inflammatory cytokines such as interleukin 1β (IL-1β). Mechanistically, lactate, at its physiological concentration, fosters the activation of inflammatory macrophages by directly binding to the catalytic domain of prolyl hydroxylase domain-containing 2 (PHD2) in a competitive manner with α-ketoglutarate and stabilizes hypoxia inducible factor (HIF-1α). Lactate-induced IL-1β was abolished in PHD2-deficient macrophages. Human adipose lactate level is positively linked with local inflammatory features and insulin resistance index independent of the body mass index (BMI). Our study shows a critical function of adipocyte-derived lactate in promoting the pro-inflammatory microenvironment in adipose and identifies PHD2 as a direct sensor of lactate, which functions to connect chronic inflammation and energy metabolism.

## Abstract (web summary)

Adipocyte tissue macrophages (ATM) are recruited and activated in obesity. The authors show that adipocytes release lactate as a signal of inflammation and that this metabolite can enhance obesity associated inflammation through stimulation of ATM by direct binding with PHD2.

## Introduction

In response to excess calorie intake, white adipose tissues are among the first to sense the alteration in nutrition status in the body. In return, they undergo extensive remodelling, leading to a chronic low grade pro-inflammatory microenvironment within the adipose tissues, termed “meta-inflammation”, which underlies the aetiology of an array of obesity-associated metabolic comorbidities^1^. Macrophages are among the most abundant immune cells in the adipose tissue of obese individuals and integral to obesity-evoked chronic inflammation^2^. Upon high calorie diet, adipose tissue macrophages (ATM) are recruited from circulation, mostly forming distinctive crown-like structures (CLS) surrounding the dying or dead adipocytes and are strongly linked with adipose inflammation and insulin resistance^3–5^. Different from the adipose resident macrophages, which are M2-like in phenotype, infiltrating macrophages are mainly CD11c^+^ pro-inflammatory, M1-like subtype and high in expression of pro-inflammatory cytokines such as interleukin-1β (IL-1β) and tumor necrosis factor (TNF), and thereby lead to an unresolved inflammation in obese adipose tissue^5,6^. Adoptive transfer and tracking experiment has shown that infiltrating monocytes/macrophages progressively polarize to the CD11c^+^ inflammatory state in obese recipients, regardless of the source of the donor monocytes, indicating that macrophage polarization is primarily determined by the microenvironment of the adipose tissue^7^. However, the exact identity of the local cue(s) contributing to the ATM polarization awaits to be uncovered.

In addition to adipokines and cytokines, metabolites present in the adipose milieu are emerging as signalling molecules that dictate the immune responses^8,9^. Lactate is the end product of anaerobic or aerobic glycolysis converted from pyruvate by lactate dehydrogenase (LDH). It has been observed that lactate production is an important metabolic feature of adipocytes. Krycer et al. showed in cultured and primary mammalian adipocytes that a substantial proportion of glucose is converted to lactate even in the presence of oxygen, similar to the Warburg effect typically seen in tumor cells^10^. Despite its abundancy in adipocytes and adipose tissue, for quite a long time lactate was simply regarded as a byproduct of glycolysis or fuel source (via Cori cycle).

The view that lactate is a metabolic waste of highly glycolytic cells has been challenged. Lactate produced by tumor cells, apoptotic cells and in inflamed tissues has been implicated in pathological conditions such as tumor growth, rheumatoid arthritis, viral infection and atherosclerotic plaque by modulating tumor associated- and muscle-macrophage polarization, CD4^+^ T cell mobility, type I interferon production and efferocytosis by apoptotic cells^11–17^. However the role of lactate in adipose tissue, especially during changes in energy metabolism, remains unclear.

Here we demonstrate that during the onset of obesity, adipocyte-derived lactate functions as a paracrine signal to potentiate the polarization of ATMs to an inflammatory state. Depletion of lactate production in adipocytes ameliorates adipose tissue inflammation and obesity-associated insulin resistance. We show that lactate directly binds with and inhibits the activity of prolyl hydroxylase domain-containing 2 (PHD2), the enzyme that hydroxylates hypoxia inducible factor 1α (HIF-1α). Lactate-evoked stabilization of HIF-1α transactivates IL-1β in the presence of inflammatory stimuli. The pro-inflammatory function of lactate is further corroborated by our observation in human omental adipose samples, showing that individuals with higher adipose lactate level is associated with more severe adipose inflammation and insulin resistance, independent of body mass index (BMI). Our study thus implicates a lactate-mediated mechanism that links metabolism and inflammation in adipose tissue during nutritional overload.

## Results

### Lactate production is selectively increased in adipocytes of obese adipose tissue

**Fig. 1:**
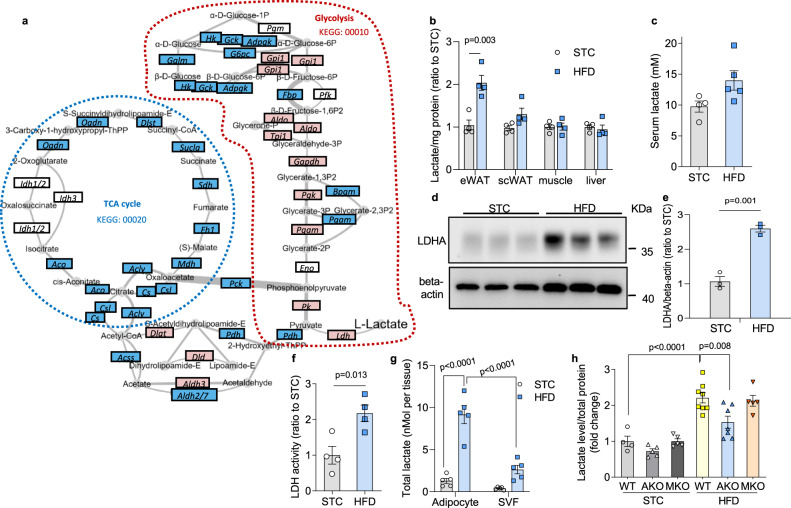
Lactate production in adipocyte is increased in diet-induced obesity. **a** Gene expression changes in glucose metabolic pathways (KEGG ID mmu00010 and mmu00020) in epididymal white adipose tissue (eWAT) of C57BL/6 mice fed with low or high fat diet for 3 months. Original data were obtained from GSE91067 (GSM2420432 - GSM2420439). Genes significantly up- or downregulated in obese eWAT were highlighted in pink (Up) and blue (Down). Width of the edge is proportional to the fold change of the gene. **b**–**h** 8-week-old male C57BL/6J mice were fed with standard chow (STC) or high fat diet (HFD) for 3 months. **b**–**c** Lactate levels in **b** different tissues (*n* = 4 biologically independent animals) and in **c** serum (STC: *n* = 4; HFD: *n* = 5 biologically independent animals) of mice. **d** Western blotting of LDHA in mouse eWAT. **e** Densitometry quantification of LDHA level by Western blotting. *n* = 3 biologically independent samples. **f** LDH activity in mouse eWAT. *n* = 4 biologically independent animals. **g** The amount of lactate in mature adipocytes and stromal vascular fraction (SVF) isolated from the whole eWAT fat pad. *n* = 5 biologically independent animals. **h** Lactate level in eWAT of wildtype (WT), adipocyte (AKO) and myeloid cell-specific *Ldha* knockout (MKO) mice. STC-WT: *n* = 4; STC-AKO: *n* = 5; STC-MKO: *n* = 5; HFD-WT: *n* = 8; HFD-AKO: *n* = 7; HFD-MKO: *n* = 5 biologically independent animals. Data represent mean ± SEM; Significance was calculated by two-way ANOVA with post hoc Bonferroni correction (**b**, **g**, **h**) or two-tailed student’s *t* test (**c**, **e**, **f**).

To further dissect the alterations in glucose metabolism in white adipose tissue (WAT) upon diet-induced obesity, genes involved in glucose metabolism, i.e. in glycolysis (KEGG ID mmu00010) and TCA cycle (KEGG ID mmu00020) were selected and re-analysed using RNASeq database on mouse epididymal white adipose tissue (eWAT) with either low fat or high fat feeding^18^ (Fig. 1a). In obese eWAT, most of the genes involved in glycolysis were upregulated, while those in TCA cycle were found downregulated (Fig. 1a). We therefore postulated that lactate production shall be elevated in eWAT after diet-induced obesity. To test this hypothesis, we measured the lactate levels in C57BL/6J mice fed with either standard chow (STC) or high fat diet (HFD) for 3 months. The results showed that lactate level was specifically elevated in the eWAT of obese mice, but not in other tissues examined, including subcutaneous WAT (scWAT), liver, skeletal muscle and in serum (Fig. 1b, c). Expression of lactate dehydrogenase A (LDHA), the predominant LDH isoform in adipose tissue (Supplementary Fig. 1), was significantly upregulated in eWAT of the obese mice (Fig. 1d, e and Supplementary Fig. 1a), which was consistent with an increment in LDH activity (Fig. 1f). In contrast, no obvious increase of LDHA expression was observed in obese scWAT (Supplementary Fig. 2). Furthermore, we found the amount of lactate was sharply induced in mature adipocytes while this trend was modest in the stromal vascular fraction (SVF) (Fig. 1g). In particular, 79% of total adipose lactate was contributed by the mature adipocytes in obese eWAT, demonstrating that adipocyte is the primary source of lactate especially in obese condition (Fig. 1g). This notion was corroborated by our finding that the lactate level in adipose tissue was profoundly reduced by adipocyte-selective deletion of *Ldha* gene (AKO) (Fig. 1h, Supplementary Fig. 3a–c). In contrast, no obvious difference in adipose lactate concentration was observed between wildtype (WT) and myeloid cell-specific *Ldha* KO (MKO) mice (Fig. 1h and Supplementary Fig. 3d), implying that overproduction of lactate in obese adipose tissue was predominantly contributed by mature adipocytes.

### Adipocyte-specific *Ldha* deletion protects mice against diet-induced insulin resistance

**Fig. 2:**
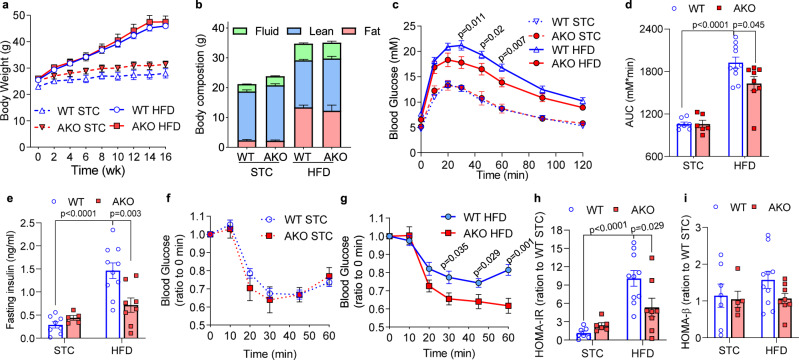
Adipocyte-specific deletion of *Ldha* protects against glucose intolerance and insulin resistance in obese mice. Wildtype (WT) and adipocyte-specific *Ldha* knockout (AKO) mice were fed with standard chow (STC) or high fat diet (HFD) for 4 months. **a** Body weight of the mice. WT-STC: *n* = 6; WT-HFD: *n* = 5; AKO-STC: *n* = 7; AKO-HFD: *n* = 9 biologically independent animals. **b** Body composition of the mice at the end of the treatment. STC-WT: *n* = 6; STC-AKO: *n* = 5; HFD-WT: *n* = 7; HFD-AKO: *n* = 5 biologically independent animals. **c** Glucose tolerance test (GTT) and **d** Area under the curve (AUC) of the GTT. STC-WT: *n* = 7; STC-AKO: *n* = 4; HFD-WT: *n* = 12; HFD-AKO: *n* = 8 biologically independent animals. **e** Fasting serum insulin level. STC-WT: *n* = 8; STC-AKO: *n* = 5; HFD-WT: *n* = 10; HFD-AKO: *n* = 8 biologically independent animals. **f**, **g** Insulin tolerance test (ITT). WT-STC: *n* = 7; WT-AKO: *n* = 4; WT-HFD: *n* = 7; AKO-HFD: *n* = 4 biologically independent animals. **h** HOMA-IR and **i** HOMA-β of the mice. WT-STC: *n* = 7; WT-AKO: *n* = 4; WT-HFD: *n* = 7; AKO-HFD: *n* = 4 biologically independent animals. Data represent mean ± SEM; Significance was calculated by two-way ANOVA with post hoc Bonferroni correction (**c-i**).

To investigate the physiological function of lactate in diet-induced obesity, 8-week-old male WT and AKO mice were fed with STC or HFD. Mice of both genotypes had comparable body weight, body composition and adipose tissue weights (Fig. 2a, b and Supplementary Fig. 3e–g). Interestingly, HFD-evoked glucose intolerance was significantly mitigated in AKO mice, as determined by glucose tolerance test (GTT) (Fig. 2c, d). This was accompanied by a reduction in fasting insulin level in obese AKO mice (Fig. 2e), suggesting that adipocyte-restricted depletion of lactate protects mice from HFD-evoked insulin resistance. This concept was confirmed by insulin tolerance test (ITT), which showed a greater glucose lowering effect of insulin in the obese AKO mice, compared to the WT mice with the same treatment (Fig. 2f, g). Consistently, HOMA-IR, an index for insulin resistance, was obviously lower in HFD-fed AKO mice, compared to their WT littermates (Fig. 2h), while there was no significant difference in HOMA-β, the index for β cell functions (Fig. 2i). In contrast, myeloid-specific deletion of *Ldha* caused a modest increase in body fat as well as exacerbation of glucose disposal (Supplementary Fig. 4a-d), while no significant difference in insulin sensitivity was observed (Supplementary Fig. 4e). Collectively these data demonstrate that adipocyte-derived lactate is causally linked to obesity-induced insulin resistance and glucose intolerance.

### Obesity-induced ATM polarization to the inflammatory subtype is ameliorated by adipocyte-selective lactate depletion

**Fig. 3:**
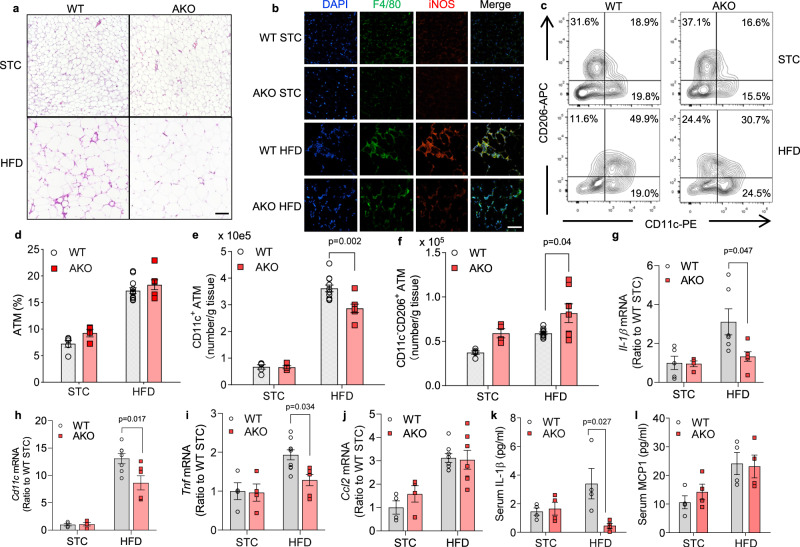
Obesity-induced adipose inflammation is attenuated by lactate depletion in adipocytes. Wildtype (WT) and adipocyte-specific *Ldha* knockout (AKO) mice were fed with standard chow (STC) or high fat diet (HFD) for 4 months before epididymal white adipose tissue (eWAT) was isolated for analysis. **a** HE staining of eWAT; scale bar, 100 μm. **b** Immunofluorescence staining of iNOS and F4/80 in eWAT; scale bar, 100 μm. **c**–**f** SVF in eWAT was subjected to flowcytometry analysis for macrophage subtypes. STC-WT: *n* = 5 (**c**–**f**); STC-AKO: *n* = 4 (**c–f**); HFD-WT: *n* = 12 (**d**) or *n* = 11(**e**) or *n* = 10 (**f**); HFD-AKO: *n* = 7 (**c**–**f**) biologically independent animals. **g**–**j** mRNA expression of **g** *Il-1β* (STC-WT: *n* = 5; STC-AKO: *n* = 4; HFD-WT: *n* = 6; HFD-AKO: *n* = 6 biologically independent animals), **h** *Cd11c* (STC-WT: *n* = 4; STC-AKO: *n* = 4; HFD-WT: *n* = 7; HFD-AKO: *n* = 6 biologically independent animals), **i** *Tnf* (STC-WT: *n* = 4; STC-AKO: *n* = 4; HFD-WT: *n* = 9; HFD-AKO: *n* = 6 biologically independent animals) and **j** *Ccl12* (STC-WT: *n* = 4; STC-AKO: *n* = 4; HFD-WT: *n* = 8; HFD-AKO: *n* = 7 biologically independent samples) in eWAT. **k**–**l** Serum levels of **k** IL-1β and **l** MCP1. *n* = 4 biologically independent animals. Data represent mean ± SEM; Significance was calculated by two-way ANOVA with post hoc Bonferroni correction (**d-i**).

The biology of the mice eWAT was further examined. As shown in Fig. 3a, after HFD feeding eWAT of the WT mice was more enriched in CLS (Fig. 3a), which is mainly composed of pro-inflammatory ATMs and correlates with insulin resistance^6^. Consistently, immunofluorescence staining showed reduced number of inflammatory ATMs (iNOS^+^ F4/80^+^) in eWAT of obese AKO mice (Fig. 3b), indicating that lactate-deficiency in adipocytes antagonizes the pro-inflammatory remodelling of ATMs. This was further supported by flow cytometry analysis showing that albeit the total number of ATMs (F4/80^+^ CD11b^+^) was indistinguishable between WT and AKO mice (Fig. 3d and Supplementary Fig. 5), the percentage of CD11c^+^ ATMs was much higher in WT mice, compared to those in AKO mice (Fig. 3c, e and Supplementary Fig. 5). In contrast, obese AKO mice had a higher number of CD11c^−^ CD206^+^ anti-inflammatory ATMs (Fig. 3f). These data demonstrate that adipocyte lactate shifts the ATMs to a more pro-inflammatory profile especially in obese condition. mRNA expression of the genes linked to M1 macrophages, including *Cd11c, Il-1β, Tnf*, was significantly lower in eWAT of the obese AKO mice (Fig. 3g–i), while the chemokine *Ccl2* remained unchanged (Fig. 3j). Additionally, serum level of IL-1β was elevated in obese WT mice, while this trend was abolished when *Ldha* was deleted in adipocytes (Fig. 3k). No difference in serum level of MCP1 was observed (Fig. 3l). These alterations observed in eWAT of obese AKO mice were absent in MKO mice fed with the same diet (Supplementary Fig. 6), which confirm that at the physiological setting, lactate produced by the mature adipocytes determines the local micro-environment in adipose and thus dictates adipose macrophage phenotypes.

### Adipocyte-derived lactate potentiates IL-1β expression in M1 macrophages

**Fig. 4:**
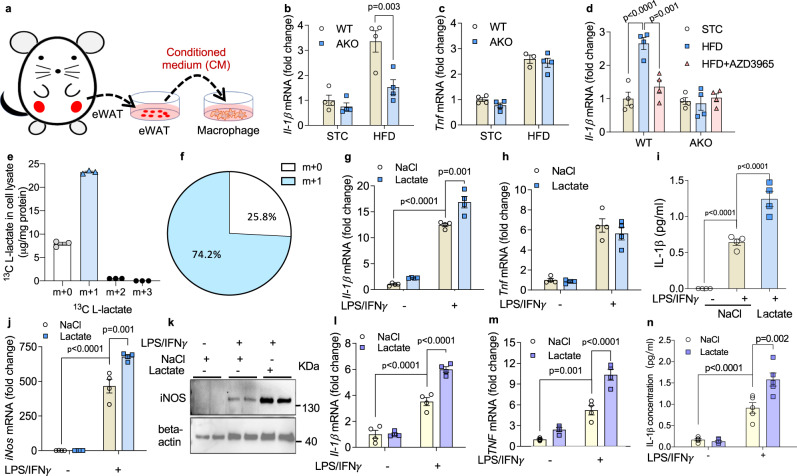
Lactate potentiates IL-1β expression in mouse and human inflammatory macrophage. **a**–**d** Conditioned medium (CM) of epididymal white adipose tissue (eWAT) was collected from wildtype (WT) and adipocyte-specific *Ldha* knockout (AKO) mice. Bone marrow derived macrophages (BMDM) were cocultured in CM for 24 hr. **a** Illustration of the co-culture experiment. **b**, **c** BMDM mRNA expression of **b** *Il-1β*, n = 4 biologically independent animals, and **c** *Tnf*, STC-WT: *n* = 4; STC-AKO: *n* = 3; HFD-WT: *n* = 3; HFD-AKO: *n* = 4 biologically independent animals. **d** *Il-1β* mRNA in BMDMs cocultured in CM from WT and AKO eWAT. One group of BMDM was cocultured in CM from obese eWAT with AZD3965 (100 nM). *n* = 4 biologically independent samples. **e**–**i** Unelicited or inflammatory BMDMs were treated with 20 mM lactate or 20 mM NaCl as control in vitro. **e**–**f** Lactate content in cell lysate was measured after BMDM was cultured with medium containing 20 mM C3-^13^C lactate for 24 hr. **e** Lactate containing zero, 1, 2 and 3 ^13^Carbons (m + 0, m + 1, m + 2, m + 3) in cell lysate. *n* = 3 biologically independent samples. **f** Relative enrichment of endogenous and m + 1 ^13^C lactate in cell lysate. **g**, **h** mRNA level of **g** *Il-1β* and **h** *Tnf*. *n* = 4 biologically independent samples. **i** Concentration of IL-1β in BMDM medium. *n* = 4 biologically independent samples. **j** mRNA level of *iNOS*. *n* = 4 biologically independent samples. **k** Western blotting of iNOS. **l**–**n** Human CD14^+^ monocytes were differentiated to macrophages and treated with 20 mM lactate or 20 mM NaCl as control. mRNA levels of **l** *IL-1β* and **m** *TNF* were examined by real time PCR. *n* = 4 biologically independent samples. **n** IL-1β concentration in medium. *n* = 5 biologically independent samples. Data represent mean ± SEM; Significance was calculated by two-way ANOVA with post hoc Bonferroni correction (**b**–**d**, **g**–**j**, **l**–**n**).

To study the action of adipocyte-produced lactate on macrophage biology, conditioned medium (CM) from lean and obese eWAT was used to treat the unelicited BMDMs (M0) from lean mice (Fig. 4a). Compared to the CM from lean mice, expression of *Il-1β* and *Tnf* in macrophage was upregulated upon culturing in obese eWAT CM (Fig. 4b, c), mimicking the physiological micro-environment in lean and obese adipose tissues. More importantly, induction of *Il-1β* by obese eWAT was almost completely abolished when macrophages were cultured in CM from AKO mice (Fig. 4b), although the mRNA level of *Tnf* was not affected in this setting (Fig. 4c). Likewise, *Ldha* KO macrophage cultured in CM from WT and AKO eWAT exhibited a similar trend of *Il-1β* expression as WT macrophage (Supplementary Fig. 7), indicating that endogenously produced lactate does not influence IL-1β expression in macrophages, which is consistent with our findings in animal studies (Fig. 3 and Supplementary Fig. 6). Notably, pharmacological inhibition of MCT1, the plasma membrane lactate transporter^19^, significantly reversed the induction of *Il-1β* by CM from eWAT of obese WT mice, but not AKO mice (Fig. 4d). In contrast, inhibition of GPR81 did not abolish the inductive effect of the obese eWAT CM on *Il-1β* (Supplementary Fig. 8). Furthermore, after incubation with the ^13^C-labled lactate in the culture medium, over 74% of the total lactate within the BMDMs were ^13^C-labled (Fig. 4e, f), implying that macrophages actively take up lactate from outside, which constitutes a significant portion of the intracellular lactate. Collectively these results demonstrate that lactate from obese adipose tissue directly controls inflammatory responses in macrophages via a process that requires the transport of lactate inside the macrophage.

Due to the heterogeneity of the macrophages, lactate likely exerts distinct actions in different sub-class of macrophages. Therefore, both unelicited (M0) and inflammatory macrophages were treated with physiological concentration of lactate in vitro (Fig. 1c)^12^. Consistent with Colegio et al., we found that in M0 macrophages, lactate mainly increased the expression level of M2-like macrophage markers such as *Arg1* (Supplementary Fig. 9), while the expression of *Il-1β* was not altered (Fig. 4g). However in inflammatory macrophages, lactate substantially stimulated the transcription of *Il-1β*, but with little effect on the expression of *Tnf* (Fig. 4g, h). Likewise, the protein concentration of IL-1β in supernatant of inflammatory macrophages was almost doubled upon lactate treatment (Fig. 4i). The activity of caspase-1 was unaltered by lactate under this condition (Supplementary Fig. 10). Moreover, both the mRNA and protein levels of iNOS, the key marker gene in inflammatory macrophage, were upregulated by lactate in inflammatory macrophages (Fig. 4j, k). Likewise, when the unelicited macrophages were incubated with the apoptotic cells, the mRNA expression of *Il-1β* was also elevated by lactate (Supplementary Fig. 11a). The action of lactate was also tested in primary human CD14^+^ macrophages, showing that addition of lactate in culture medium led to elevated levels of *IL-1β* and *TNF* (Fig. 4l–n). These data indicate that lactate boosts inflammatory responses specifically in the pro-inflammatory macrophages. Meanwhile, we examined the key metabolites in glycolysis and TCA cycle in pro-inflammatory macrophages with or without lactate treatment. It was shown that despite abundant elevation in intracellular lactate level, the key metabolites in glycolysis and TCA cycle were not significantly altered, except for succinate which was significantly reduced after lactate treatment (Supplementary Fig. 12 and Supplementary Data 1).

### Lactate increases HIF-1α protein in macrophages

**Fig. 5:**
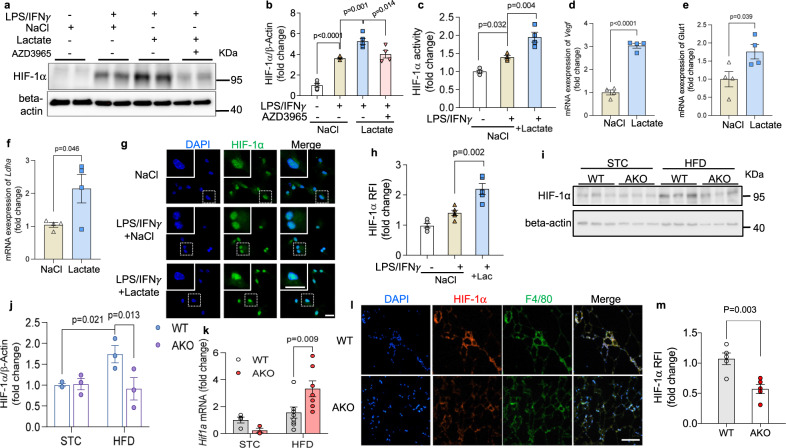
HIF-1α protein level is increased by lactate in pro-inflammatory macrophages. **a**–**h** Mouse BMDMs were treated with lactate (20 mM) or NaCl (20 mM) and AZD3965 (100 nM). **a** Western blotting of HIF-1α in macrophages. **b** Densitometry quantification of HIF-1α level by Western blotting. **c** HIF-1α activity in macrophages. M0: *n* = 5; M1: *n* = 5; M1 + Lac: *n* = 5; M1 + Lac + MCTi: *n* = 4 biologically independent samples. **d**–**f** mRNA expressions of HIF-1α downstream genes. *n* = 4 biologically independent samples. **g** Immunofluorescence staining of HIF-1α in BMDMs with lactate or NaCl treatment; scale bar, 20 μm. **h** Relative fluorescence intensity of HIF-1α in **g**. *n* = 5 biologically independent samples. **i** Western blotting of HIF-1α in eWAT of lean and obese WT and AKO mice. **j** Densitometry quantification of HIF-1α level in **(i)**. n = 3 biologically independent samples. **k** mRNA level of *Hif1a* in epididymal white adipose tissue (eWAT). STC-WT: *n* = 4; STC-AKO: *n* = 3; HFD-WT: *n* = 10; HFD-AKO: *n* = 7 biologically independent animals. **l** Immunofluorescence staining of HIF-1α and F4/80 in eWAT; scale bar, 100 μm. **m** Relative fluorescence intensity of HIF-1α in (**l**). *n* = 5 biologically independent samples. Data represent mean ± SEM; Significance was calculated by two-way ANOVA with post hoc Bonferroni correction (**b**, **c**, **h**, **j**, **k**) or two-tailed student’s *t* test (**d**–**f**, **m**).

Our findings coincided with the work by Tannahill et al. that HIF-1α controls the transcription of *Il-1β* but not *Tnf* or *iNos* in mouse BMDMs^20^. We therefore tested whether lactate modulates the activity of M1 macrophage through HIF-1α. Western blotting showed that lactate increased HIF-1α protein level in inflammatory macrophages (Fig. 5a, b). Likewise, compared to the M0 macrophages, the transactivation activity of HIF-1α was significantly increased in inflammatory macrophages, which was further potentiated upon addition of lactate (Fig. 5c). Consistently, the mRNA expression of HIF-1α downstream genes, including *Vegf*, *Glut1* and *Ldha* was increased upon lactate treatment in inflammatory macrophages (Fig. 5d–f). As indicated by immunofluorescence staining, HIF-1α expression was relatively low and in a diffused state in the quiescent M0 macrophages. In inflammatory macrophages, HIF-1α was upregulated and more densely-localized in the nuclei, and this trend was further boosted by supplementation of lactate (Fig. 5g, h). Similarly when the macrophages were incubated with the apoptotic cells, HIF-1α expression was enhanced by lactate at the protein level but not at the mRNA level (Supplementary Fig. 11b, c). In eWAT the protein level of HIF-1α in obese AKO mice was much lower than that in matched WT mice (Fig. 5i, j). Furthermore, by immunostaining, we found that HIF-1α was mainly present in F4/80^+^ ATMs, and its expression was less prominent in AKO mice (Fig. 5l, m). In contrast, the mRNA level of *Hif1a* was elevated in obese AKO mice (Fig. 5k), suggesting that lactate-associated increment of HIF-1α is post-transcriptionally regulated.

### Lactate directly targets and inhibits PDH2 activity to stabilize HIF-1α

**Fig. 6:**
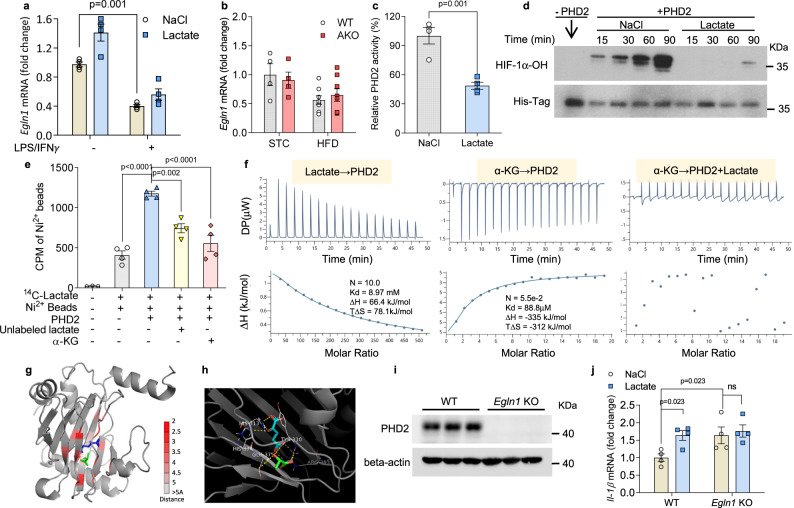
Lactate directly binds and inhibits the activity of PHD2. **a** *Egln1* mRNA level in macrophages with 20 mM lactate or NaCl. *n* = 4 biologically independent samples. **b** mRNA of *Egln1* in epididymal white adipose tissue (eWAT) of wildtype (WT) and adipocyte-specific *Ldha* knockout (AKO) mice. STC-WT: *n* = 4; STC-AKO: *n* = 4; HFD-WT: *n* = 8; HFD-AKO: *n* = 8 biologically independent animals. c In vitro fluorometric PHD2 activity assay in the presence of 20 mM lactate or NaCl. *n* = 4 biologically independent samples. **d** His-tagged HIF-1α was incubated with PHD2 in presence of 20 mM lactate or NaCl. Time-dependent HIF-1α hydroxylation was examined by Western blotting. **e** Pull down assay between PHD2 and lactate. His-tagged PHD2 protein was incubated with ^14^C-labeled lactate. Unlabeled lactate or α-ketoglutarate (α-KG) was added where indicated. Count per min (CPM) in Ni-NTA beads was measured by liquid scintillation counter. Group 1: *n* = 3; Group 2,3,4,5: *n* = 4 biologically independent samples. **f** Isothermal titration calorimetry (ITC) of PHD2 with lactate and α-KG. Sequential heat pulses for each injection (upper panel) and the integrated data (lower panels). α-KG → PHD2: injecting α-KG to PHD2; Lac→PHD2: injecting lactate to PHD2; α-KG → PHD2 + Lactate: injecting α-KG to PHD2 + lactate mixture. **g**, **h** Representative images of autodocking for catalytic domain of PHD2, lactate and α-KG. Blue: α-KG, Green: lactate, yellow dash lines: hydrogen bonds. **i**, **j** *Egln1* gene was knocked out by Crispr/Cas9 mediated method in immortalized mouse BMDM. The macrophages were treated with lactate or vehicle control. **i** Western blotting of PHD2 in WT and *Egln1* KO macrophage. **j** mRNA level of *Il-1β* in WT and *Egln1* KO macrophages with 20 mM lactate or NaCl. *n* = 4 biologically independent samples. Data represent mean ± SEM; Significance was calculated by two-way ANOVA with post hoc Bonferroni correction (**a**, **b**, **e**, **j**) or two-tailed student’s *t* test (**c**).

PHD2, encoded by *Egln1*, is the enzyme that hydroxylates and destabilizes HIF-1α in macrophage^21^. We then investigated whether lactate impacts the expression and activity of PHD2. In in vitro cultured BMDMs, although *Egln1* mRNA was suppressed in inflammatory macrophages, lactate treatment did not alter its expression (Fig. 6a). Likewise, the mRNA level of *Egnl1* in eWAT was comparable between WT and AKO mice (Fig. 6b). We thus sought to examine whether lactate influences the hydroxylase activity of PHD2. To this end, recombinant protein containing the catalytic domain of human PHD2 (PHD2_181–426_) and its substrate, the oxygen dependent degradation domain (ODDD) of HIF-1α (HIF-1α_401-603_) were used to establish the in vitro PHD2 activity assay (Supplementary Fig. 13)^22,23^. We demonstrated that the physiological concentration of lactate exerted potent inhibition against the hydroxylation activity of PHD2 on HIF-1α (Fig. 6c). Consistently Western blotting of hydroxylated HIF-1α showed that PHD2-induced HIF-1α hydroxylation was profoundly diminished in the presence of lactate (Fig. 6d). Furthermore, by in vitro radioligand binding assay, the direct interaction between PHD2 protein and ^14^C-labeled lactate was readily detected, and the binding was compromised by competitively adding the unlabelled lactate or α-ketoglutarate (α-KG), the endogenous substrate of PHD2 (Fig. 6e). The binding between PHD2 and lactate was quantitatively determined by isothermal titration calorimetry (ITC) (Fig. 6f). Moreover, the binding between PHD2 and α-KG was diminished when PHD2 was pre-incubated with lactate (Fig. 6f). These results demonstrate lactate binds at the catalytic domain of PHD2 that blocks the access of its natural substrate α-KG, and hence inhibits the activity of PDH2. Molecular docking was performed to assess the possible binding sites of lactate on the catalytic domain of PHD2. The predicted binding mode of lactate to PHD2 revealed a good shape match between lactate and the binding pocket (Fig. 6g); Residues Tyr310 and Glu375 of PHD2 forms hydrogen bonds with lactate (Fig. 6h). Although the predicted binding site of lactate does not completely overlap with that of α-KG, distance analysis indicates that AA309-313 and AA374-378 of PHD2 interact with lactate, among which Arg252, Asp254, Tyr310 and Val376 are also involved in the interaction with α-KG (Supplementary Fig. 14), and therefore possibly hinders the binding between α-KG and PHD2.

To functionally validate the link between lactate and PHD2, we generated *Egln1* gene KO macrophages by Crispr/Cas9-based method (Fig. 6i). We found that in PHD2 deficient inflammatory macrophages, lactate-evoked induction of *Il-1β* was lost (Fig. 6j), demonstrating that PHD2 indeed serves as a mediator to facilitate the pro-inflammatory action of lactate in inflammatory macrophages.

### Adipose lactate is correlated with insulin resistance and adipose inflammation

**Fig. 7:**
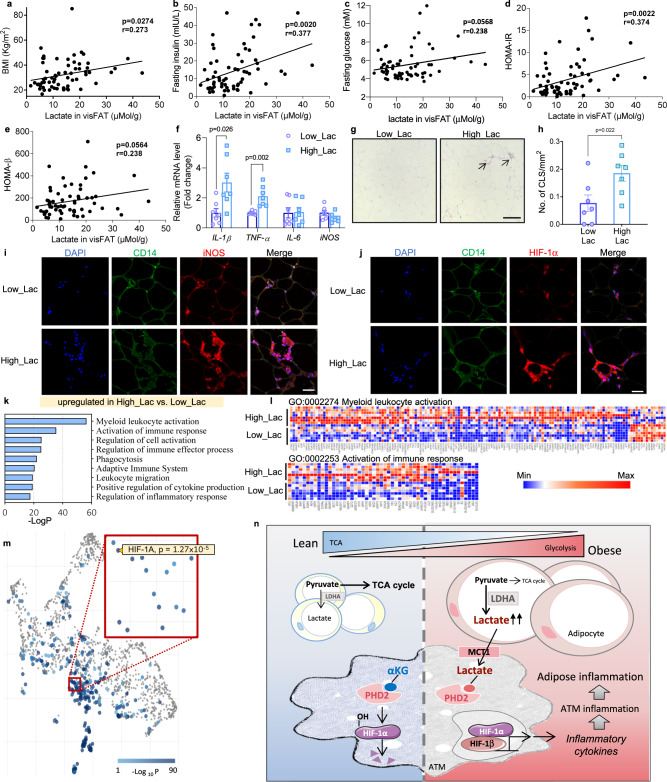
Human adipose lactate level is positively correlated with adipose inflammation and insulin resistance independent of BMI. **a**–**e** Correlation between lactate levels in human omental adipose tissues with **a** BMI, **b** fasting insulin, **c** fasting glucose, **d** HOMA-IR and **e** HOMA-β index. *n* = 65 subjects. **f**–**m** 7 omental fat samples were randomly selected from low and high lactate groups (Low_Lac vs. High_Lac). **f** Inflammatory cytokines mRNA level in adipose. *n* = 7 individuals. **g** HE staining of omental fat; scale bar, 200 μm. Arrows point at the crown like structures (CLSs). **h** CLS numbers in omental fat. *n* = 7 individuals. **i**, **j** Immunofluorescence staining of iNOS and HIF-1α and human macrophage marker CD14; scale bar, 40 μm. **k**, **l** RNASeq of omental fat (*n* = 7 individuals). **k** GO enrichment of upregulated pathways in High_Lac group. **l** Heatmap showing differentially expressed genes in GO:0002274 and GO:0002253. **m** Transcription factor (TF) enrichment in upregulated genes in High_Lac group. Significantly enriched TFs (*p* < 0.05) were highlighted in blue. HIF-1α was shown in the enlarged region. **n** Illustration of the study hypothesis. Data represent mean ± SEM; Statistics were performed using spearman correlation analysis (**a**–**e**), two-way ANOVA with post hoc Bonferroni correction (**f**) or two-tailed student’s *t* test (**h**).

To examine the potential involvement of lactate in human subjects, we collected 65 omental adipose tissues, which is the typical white adipose tissue in the human abdomen^24^, from patients undergoing elective abdominal surgery (Supplementary table 1). The lactate concentration within the omental fat tissues positively correlated with BMI, fasting insulin and HOMA-IR (Fig. 7a–e). Furthermore, partial correlation analysis demonstrated that adipose lactate level still remained positively associated with fasting insulin (*r* = 0.273, *p* = 0.029) and HOMA-IR (*r* = 0.277, *p* = 0.027), after adjustment for BMI (Table 1), indicating adipose tissue lactate is linked to insulin resistance in a manner independent of BMI. Furthermore, we divided these samples into high lactate and low lactate groups (median lactate concentration = 13.96 mMol/g protein). 7 samples were randomly picked from each group for further analysis. Real time PCR analysis showed that *IL-1β* and *TNF* were significantly higher in the adipose tissues with higher level of lactate (Fig. 7f), which is consistent with our findings in animal models. Moreover, we quantified the number of CLSs in adipose depots and it was shown that individuals with higher adipose lactate had greater number of CLSs, compared to those with lower level of local lactate content (Fig. 7g, h). Notably, inflammatory macrophage marker iNOS as well as HIF-1α in ATMs were expressed at a much higher level in those in high lactate group (Fig. 7i, j). These adipose samples were further subjected to bulk RNASeq analysis. Pathway enrichment analysis revealed that in high lactate adipose tissue, pathways involved in myeloid activation and immune responses are engaged (Fig. 7k, l), further corroborating our hypothesis that lactate concentration is positively and strongly associated with pro-inflammatory responses in ATMs. Furthermore, transcription factor enrichment analysis identified several transcription factors that are differentially implicated between low and high lactate groups. Within them HIF-1α was identified as a hit (Fig. 7m and Supplementary table 2), which corroborates our hypothesis.

**Table 1:** Correlation between human adipose tissue lactate concentration with clinical parameters

		Correlation	Partial correlation (controlled by BMI)
Insulin	Correlation	0.377	0.273
	Significance (2-tailed)	0.002	0.029
	df	63	62
HOMA IR	Correlation	0.374	0.277
	Significance (2-tailed)	0.002	0.027
	df	63	62
HOMA beta	Correlation	0.238	0.136
	Significance (2-tailed)	0.0564	0.284
	df	63	62
Glucose	Correlation	0.238	0.172
	Significance (2-tailed)	0.0568	0.175
	df	63	62
BMI	Correlation	0.273	–
	Significance (2-tailed)	0.027	–
	df	63	–

## Discussion

Prior work demonstrated that serum lactate level is elevated among obese, hypertensive and insulin-resistant subjects^25,26^ and correlates with both fasting glucose and glycated haemoglobin concentrations and type 2 diabetes^26^. One prospective study established serum lactate as an independent risk factor for type 2 diabetes^27^. Lee et al. reported that lactate level was acutely elevated in mouse adipose tissue after high fat diet feeding^28^. Despite these early findings on the positive associations between lactate and metabolic disorders, lactate is still mainly used as a measure of hypoxia or impaired oxidative capacity while little attention is drawn to the pathological impact of lactate on metabolic diseases and adipose tissue inflammation. In the current study, we found that adipocyte is the major source of lactate in white adipose tissue, by fractionation of adipose tissue and cell type specific *Ldha* KO mice respectively. Interestingly, a recent study showed that lactate production is prioritized in adipocytes, independent of glucose metabolism^10^, indicative of the additional regulatory role of lactate beyond lipid synthesis and storage in adipocytes. Indeed, lactate has been identified as a browning inducer in both murine and human white adipocytes, via a process that requires active PPARγ signalling^29^. In addition to this autonomous action, we provide evidence that adipocyte-derived lactate functions as a bioactive signalling cue to facilitate the intercellular communication between adipocytes and ATMs to elicit pro-inflammatory responses, especially in the context of diet-induced obesity.

The action of lactate is highly variable and cell type dependent. Tumor-derived lactate suppresses the immune response against the tumor itself in tumor-associated macrophages (TAM) and T cells, thus creating a milieu permissive to the cancer cell growth^11,12,16,30^. Likewise, Zhang et al. found that endothelial lactate control muscle recovery by modulating muscle macrophage polarization, which favors angiogenesis, a condition reminiscent of tumor microenvironment^17^. Furthermore, previous work has also revealed that efferocytic phagocytes influence non-engulfing naive macrophages in the tissue microenvironment towards anti-inflammatory polarization via release of lactate as well as other factors (such as TGFβ and IL-10)^13^. In contrast, the immune response to lactate in the context of chronic or acute inflammation is quite different from that seen in the tumor or muscle. Our data are in line with Samuvel et al. who showed that lactate boosts the toll-like receptor 4 (TLR4) ligand evoked-M1 polarization in human macrophages^31^. Furthermore, lactate buildup at the site of chronic inflammation promotes rheumatoid arthritis by inducing CD4^+^ T cell metabolic rewiring, acting as a pro-inflammatory signal^14^. Therefore it is possible that lactate exerts distinct functions in modulating adipose microenvironment in lean and obese conditions where the anti-inflammatory and pro-inflammatory stimuli predominates respectively.

These seemingly contradictory observations coincided by the dual actions of HIF-1α in inflammation and macrophage polarization. In the case of TAMs which predominantly exist in M2-like states, lactate-evoked polarization of TAM to the M2 subtype was largely abolished under a HIF-1α-deficient condition^12^. On the other hand, HIF-1α has been shown to positively regulate pro-inflammatory cytokine production and promote inflammation in myeloid cells in experimental models of bacterial infection, rheumatoid arthritis, atherosclerosis, non-alcoholic steatohepatitis and obesity, conditions that favor inflammatory remodeling^32–36^. Likewise, exposure of macrophages to saturated fatty acid increased glycolysis and HIF-1α expression, which culminated in IL-1β induction^37^. Furthermore, mice with macrophage-specific deletion of HIF-1α partially mirrored the phenotypes of our obese adipocyte-selective *Ldha* KO mice, in that both mice exhibited reduced levels of local and systemic IL-1β^37^, consistent with the notion that myeloid HIF-1α activation in obese adipose tissue, where inflammatory stimuli predominate, positively drives inflammation in obesity. The discrepancy regarding the function of HIF-1α in different subtypes of macrophages warrants future investigation.

Enhanced glycolysis is associated with HIF-1α protein accumulation. Previous literature reported that lactate stabilizes HIF-1α in unstimulated macrophages but the detailed mechanism is not elucidated^12^. We found that in mice with adipocyte-restricted lactate depletion, HIF-1α was downregulated in adipose tissue and in ATMs at the protein level, despite elevated mRNA level, suggesting that lack of lactate in adipocytes mainly compromises the protein stability of HIF-1α. As a decisive element for the transcriptional regulation of genes under low oxygen conditions, HIF-1α protein is dynamically controlled under normal and hypoxic conditions. In normoxia, HIF-1α is hydroxylated on proline residues within its central oxygen-dependent degradation domain (ODDD), a reaction catalysed by 2-oxoglutarate-dependent enzymes PHD1-3, leading to subsequent ubiquitination and degradation of HIF-1α^38^. In hypoxia, hydroxylation and ubiquitination are blocked and thus HIF-1α accumulates in cells. Our in vitro assays further demonstrated that physiological concentration of lactate suppresses the hydroxylation activity of PHD2, the major PHD isoform in macrophages^21^. This coincides with a previous study in tumor cells which showed that the effect of lactate on HIF-1α expression was abolished upon siRNA-mediated knockdown of PHD2^39^. Hence overproduction of lactate likely arouses a pseudo-hypoxic response in ATMs and thereby perpetuates the local production of IL-1β. Previous work by Tannahill et al. identified succinate, which is induced by lipopolysaccharide (LPS) as a down-stream metabolite of glycolysis, to be a stabilizer of HIF-1α by succinylation of the protein^20^. But our metabolomics study showed that the glycolysis was not significantly altered by exogenous lactate supplementation, nor was TCA cycle. Furthermore succinate was reduced after lactate treatment. We are uncertain about the exact reason causing this change but it excludes the involvement of succinate-assisted HIF-1α stabilization in exogenous lactate treatment condition.

The physiological concentration of lactate in blood is around 1.5–3 mM, and in certain tissues such as tumor it exists at up to 5–30 mM, measured by quantitative bioluminescence imaging and magnetic resonance spectroscopy^40,41^. Currently there is no study to directly quantify the lactate concentration in adipose tissue. We estimated the concentration of lactate in our study; 1 gram of human adipose tissue contains 0.32–8.70 μmol lactate, which corresponds to 0.35–9.67 mM lactate (assuming the density of the adipose tissue is 0.9 g/ml^42^). This is consistent with our ITC analysis which calculated the Kd of lactate to PHD2 around 8 mM, indicating that at the physiological condition, especially in obese status, lactate is capable of binding with and inhibiting PHD2. Consistently, 6–50 mM lactate is commonly used for in vitro studies^12,16,43^.

Studies by us and others have established lactate as a key signal that mediates cell-cell communication. Although cell surface lactate receptor GPR81 has been identified^44^, in this study we demonstrate that adipocyte-derived lactate modulates the ATMs largely via its intracellular actions, since blockade of lactate transport inside the macrophage by MCT-1 inhibitor substantially abolished the induction of *Il-1β* by conditioned medium from obese adipose tissue. A study has reported that LPS and TNF treatment significantly augmented MCT-1 expression, in macrophages, accompanied by increased lactate uptake^45^. MCT-1 has been established as a therapeutic target for cancer treatment. Preclinical work has shown that the selective MCT-1 inhibitor, AZD3965, potently inhibited the tumor growth in models of Diffuse Large B-Cell Lymphoma (DLBCL) and Burkitt’s lymphoma^46^. Thus, it is intriguing to test the in vivo efficacy of MCT-1 inhibitors in the treatment of obesity-associated metabolic diseases.

It is also worth to mention that one of the most prominent difference between WT and AKO adipose tissue is that there are less CLSs in the adipose tissue of obese AKO mice, implying that the adipocytes in AKO mice are likely less apoptotic compared to the WT control. Indeed lactate deficiency would also lead to a change in adipocyte biology per se and it is currently unclear whether and how the apoptotic status of the adipocytes also contribute to the healthier metabolic phenotype in AKO mice and warrant future investigation. Furthermore, it should also be noted that in the current study, we mostly use LPS and IFN-γ to stimulate BMDM for in vitro studies. However, these stimuli are not necessarily the pro-inflammatory signals that macrophages would get in obese adipose tissues. Instead, it is possible that lactate arouses the pro-inflammatory responses in ATMs in the presence of apoptotic hypertrophic adipocytes. Indeed, when the macrophages were incubated with the apoptotic cells, lactate arouses the similar changes as in the presence of LPS and IFN-γ, indicating lactate augments macrophage inflammation and HIF1α protein stability in the presence of other physiological stimuli, such as apoptotic cells.

NLRP3 inflammasome is critical in regulating inflammation. But the effect of lactate on inflammasome activity has been inconclusive. Lactate induces phosphorylation of PKR and NLRP3 inflammasome-dependent IL-1β secretion in nigericin, ATP, monosodium urate (MSU) crystals, or alum stimulated BMDM and THP-1 cell^47,48^. However, in other studies, in macrophages and monocytes, exogenous lactate reduced TLR4-mediated induction of IL-1β, NLRP3 and pro-caspase-1^49^. We found that lactate did not significantly alter the caspase-1 activity in pro-inflammatory macrophages. One possible explanation is that the different macrophages have distinct predominant lactate receptors, which is to be elucidated in future.

We examined the NLRP3 inflammasome activity by measuring the caspase-1 activity in M1 macrophages treated with lactate or vehicle control. As shown below, the caspase-1 activity was marginally affected by lactate treatment, suggesting that despite the inhibitory role of lactate on inflammasome activity in unelicited BMDM^49^, in M1 macrophages lactate-evoked HIF1α-PHD2 axis predominates, leading to an elevated IL-1β secretion. Interestingly, we observed a robust reduction in *Gpr81* expression in M1 macrophages and we thus wonder the lowered level of *Gpr81* may possibly explain the lack of inflammasome inhibition for lactate in M1 macrophages.

In summary, the present study implicates the function of adipocyte-released lactate as a mediator in ATM polarization and adipose inflammation by directly targeting the macrophage PHD2, followed by stabilization and transactivation of HIF-1α. Our findings underscore the interplay between immunology and metabolism in dietary obesity. In addition to cell- and animal-based evidence, a positive correlation between adipose lactate and insulin resistance index has been established in clinical samples. This highlights the feasibility that therapies targeting adipocyte lactate production and/or transport may represent an alternative for management of obesity-related meta-inflammation.

## Methods

### Animals and human studies

All procedures of animals were in accordance with the research ethics guidelines for the use of laboratory animals of the Committee on the Use of Live Animals in Teaching and Research in the University of Hong Kong (CULATR No. 3967-16) and Animal Experimentation Ethics Committee in the Chinese University of Hong Kong (AEEC No. 21-051-MIS). Human study was approved by the Institutional Review Board of Jinan University (2016-017).

*Ldha*^*fl/fl*^ *Mus musculus* of C57BL/6 J background with two LoxP sites flanking the exon3 of *Ldha* gene were generated by Shanghai Model Organisms Center, Inc. The mice were mated with AdipoQ-Cre and Lysozyme2-Cre transgenic mice (Jaxson Laboratory) to obtain the adipocyte and myeloid cell specific *Ldha* KO mice. 8-week-old male mice were housed in a controlled environment (22 °C ± 1 °C and 60–70% humidity with 12 h light/dark cycle) and fed ad libitum with standard chow (LabDiet, #5053) or 45 kcal% high fat diet (Research Diet, #D12451). Body composition was monitored by the Nuclear Magnetic Resonance (NMR) analyzer (LF90II, Bruker). For human study, written informed consents were obtained from all participants before the study. No compensation was provided. Those with one of the following criteria were excluded: serious cardiovascular diseases, thyroid dysfunction, malignant tumor, daily cigarette smoking >10 /day and daily alcohol intake >40 g (male) and >20 g (female), or high-sensitive C-reactive protein level >5 mg/dl. 1 g of human omental adipose tissue was collected during the elective surgery, fixed in 4% formalin PBS solution or put in liquid nitrogen immediately and stored in −80 °C for future experiments.

### Data mining of RNA sequencing data

Original data from GSE91067 (GSM2420432 - GSM2420439) in GEO database, which contains the RNA sequencing data of the epididymal white adipose tissue of C57BL/6J mice treated with low fat diet (10% fat) or high fat diet (60% fat) were re-analysed with EdgR R package. The generated RPKM was further analysed with KEGG database and the changes in glucose metabolic pathways (KEGG ID mmu00010 and mmu00020) were mapped with Cytoscape 3.7.1.

### Measurement of lactate and LDH activity

Mouse tissue or human omental adipose tissue (~20 mg) was homogenized in RIPA lysis buffer (150 mM NaCl, 1% Nonidet P-40, 0.5% Sodium deoxycholate, 0.1% SDS, 25 mM Tris, pH=7.4). LDH activity was measured using the Liqui-UV LDH activity assay kit (Stanbio, #2940), followed by normalization with the protein concentration measured by BCA Assay kit (Thermo Fisher Scientific, #23227). For measurement of lactate levels, the supernatant of the tissue lysates were de-proteinated by going through a 3kD cut-of Centricon (Millipore). The de-proteinated fraction was then used for quantification of the lactate by the lactate assay kit (Sigma-Aldrich, #MAK064) following the instructions.

### Glucose tolerance test (GTT) and insulin tolerance test (ITT)

Mice were fasted for 16 hrs (for GTT) or 8 hrs (for ITT) before intraperitoneally injected with D-glucose (1 g/kg body weight, for GTT) or insulin (0.75 IU/kg body weight, for ITT). Blood glucose levels were monitored with the OneTouch Glucose Meter (Accu-Chek, Roche). Fasting insulin level was measured by Mouse Insulin ELISA kit (BioVendor, #RA19004R).

### Histological and immunofluorescence staining

Adipose tissues were fixed in 4% formalin PBS solution for 24 hrs, dehydrated, embedded in paraffin and sectioned at 5 μm. The sections were deparaffinized, re-hydrated and then subjected to haematoxylin and eosin staining (Sigma) or immunofluorescence staining. For immune-staining, the sections were blocked in 3% BSA (Roche), 10% FBS (Gibco), PBS, pH = 7.4 for 1 hr, followed by incubation with primary antibodies in PBST (PBS, 0.1% Tween 20) containing 3% BSA overnight at 4 °C. The next day, the slides were washed three times in PBST followed by incubation with a fluorophore-conjugated secondary antibody for 1 hr at dark. Slides were counterstained with Gold Antifade Mountant with DAPI (Thermo Fisher Scientific) and visualized with an Olympus biological microscope BX41, and images were captured with an Olympus DP72 color digital camera (for HE staining) and Carl Zeiss LSM800 inverted confocal microscope (immunofluorescence staining). The antibodies used include F4/80 (1:100, Thermo Fisher Scientific, #14-4801-85, BM8), iNOS (1:250, Thermo Fisher Scientific, #PA3-030A), HIF-1α (1:400, Proteintech, 20960-1-AP), CD14 (1:100, Biolegend, #325604, HCD14), Chicken anti-Rat IgG (1:200, Thermo Fisher Scientific, #A21470) and Goat anti-Rabbit IgG (1:200, Thermo Fisher Scientific, #A11011).

### Isolation of adipocytes and stomal vascular factions (SVF)

Adipose tissue pads were digested in 1.5 mg/ml collagenase type I (Invitrogen) in DMEM for 40 min at 37 °C in CO_2_ incubator without shaking. The digested mixture was filtered through a 100μm cell strainer (BD Biosciences) and centrifuged at 800 xg for 10 min. The upper layer (adipocytes) and the pelleted cells (SVF) were collected for further analysis.

### Quantification of ATMs and its subtypes by flow cytometry

The SVF pellets isolated from adipose tissue were washed with red blood cell lysis buffer (NH_4_Cl 150 mM, KHCO_3_ 10 mM, Na_2_EDTA 0.1 mM, pH = 7.4) and PBS before staining with antibodies or the isotype control antibodies in PBS with 3% BSA for 1 hr at 4 °C. After washing with PBS, the cells were fixed in 4% formalin and analysed on LSR Fortessa Analyzer (BD Biosciences). Antibodies used are F4/80-FITC (1:100, Biolegend, #123108, BM8), CD11b-pacific blue (1:100, Biolegend, #101224, M1/70), CD206-APC (1:100, Biolegend, #141708, C068C2) and CD11c-PE (1:100, Biolegend, #117308, N418). The lymphocyte singlets were gated and adipose macrophages were defined as F4/80^+^ CD11b^+^ cells, followed by determination of macrophage subtypes.

### Cell culture

Bone marrow derive macrophages (BMDM) were prepared as previously described^50^. Briefly, bone marrow cells were isolated from the femur and tibia of 8-week-old male mice and differentiated to mature macrophages for 7 days in 80% RPMI1640 (Gibco) supplemented with 10% FBS, 20% L929 conditioned medium. At day 7, the differentiated macrophages were polarized to M1-like inflammatory macrophage by treating with IFN-γ (100 ng/ml, Peprotech) and LPS (10 ng/ml, Sigma) for 8 hrs (for Q-PCR) or 24 hrs (for western blotting and immunofluorescence staining).

### Co-culture of eWAT and mouse BMDM

WT and AKO mice fed with STC or HFD for 1 month before the eWAT was collected and cut into small pieces (~1mm^3^). The tissue was cultured in DMEM with 1% BSA (0.1 mg tissue/ml) in 37 °C with 5% CO_2_ for 24 hrs, and the conditioned medium were filtered through a 0.22μm filter (Millex). The conditioned medium (1:3 dilution) was used to culture BMDM for 24 hrs.

### Crispr/Cas9-aided knockout of *Egln1*

Mouse BMDMs were immortalized by infected with lentivirus expressing SV40 and then transfected with Cas9 plasmid (GENEWIZ, Inc.) using polyethylenimine (Generon). The Cas9+ cells were selected with hygromycin (200 μg/ml, Sigma-Aldrich) and then transfected with gRNA plasmid targeting mouse *Egln1* (guide sequence1: 5ʹ-CGCGGCGGCCTCGCGCGTAC-3ʹ and guide sequence2: 5ʹ-TCCCGCTGCAGTGGCGGATC-3ʹ) or scramble control gRNA (guide sequence: 5ʹ-GCACTACCAGAGCTAACTCA-3ʹ) plasmid (GENEWIZ, Inc.). After selection with puromycin (2 μg/ml, Sigma-Aldrich), the positive clones were identified by sequencing.

### Isolation of human CD14^+^ monocyte and differentiation to macrophage

Peripheral blood mononuclear cells were isolated from a 29-year-old healthy man by density gradient centrifugation with ficoll (Sigma-Aldrich). CD14^+^ monocytes were isolated using Human Monocyte Isolation Kit (Stemcell Technologies Inc, #19669), cultured in DMEM 10% FBS and stimulated with 50 ng/ml of phorbol 12-myristate 13-acetate (Sigma) for 24 hrs before the treatment^51^.

### HIF-1α activity measurement

The nuclei were isolated from macrophages by swelling the cell in hypotonic buffer (20 mM HEPES pH = 7.5, 5 mM NaF, 100 μM Na_2_MoO_4_, and 1 mM EDTA) on ice for 15 min, followed by addition of Nonidet P-40 to a final concentration of 1.67%. After centrifugation at 800 xg for 10 min, the nuclei were lysed in extraction buffer (10 mM HEPES pH = 7.9, 0.1 mM EDTA, 1.5 mM MgCl2, 840 mM NaCl, 1 mM DTT, 10% glycerol) with proteinase inhibitors (Merck) by vortex on ice. The nuclei lysate was used to quantify HIF-1α activity by HIF-1 alpha Transcription Factor Assay Kit (abcam, #ab133104).

### PHD2 activity assay

The cDNA of human PHD2_181-426_ and HIF-1α_401–603_ were subcloned into pET-28a vector and the recombinant proteins with His-tag were purified from BL21 E.Coli. PHD2 activity was measured as previously described^22,23^. Briefly, for fluorometric-based Δα-KG assay, 30 μl of reaction mixture (50 mM Tris PH = 7.4, 100 mM α-KG, 150 mM FeSO_4_, 1 mM DTT, 0.6 mg/ml catalase, 1 mg/ml HIF-1α_401–603_, 130 μg/ml PHD2_181-426_ and 20 mM sodium lactate or 20 mM NaCl as control) was incubated at 37 °C for 2 hrs. Before analysis, 90 μl of 2.5 mg/ml o-Phenylenediamine (OPD) (dissolved in 0.5 M HCl) was added to the mixture and heated at 95 °C for 10 min. 60 μl of 1.25 M NaOH was added to the mixture followed by fluorescence reading with the 340 nm of excitation and 420 nm of emission. For HIF-1α hydroxylation assay, the reaction mixture (20 mM Tris pH = 7.5, 5 mM KCl, 1.5 mM MgCl_2_, 1 mM DTT, 2 mM α-KG, 2 mM ascorbate, 100 μM FeSO4, 250 μg/ml HIF-1α_401–603_, 5 μg/ml PHD2_181–426_ and 20 mM sodium lactate or 20 mM NaCl as control) was incubated at 37 °C for different time points, and the hydroxylated HIF-1α was detected with western blotting.

### PHD2 ligand binding assay

^14^C-lactate (5μCi/ml) (PerkinElmer) with or without unlabelled lactate was incubated with 25 μg/ml PHD2_181-426_ protein in the reaction buffer (20 mM Tris pH = 7.5, 5 mM KCl, 1.5 mM MgCl_2_, 1 mM DTT, 2 mM ascorbate, 100 μM FeSO4) at 37 °C for 1 hr. His-tagged PHD2_181-426_ was pulled down by Ni-NTA beads and the radioactivity of the beads was measured with liquid scintillation counter.

### Isothermal titration calorimetry

Recombinant PHD2_181-426_ and compounds (1.5 mM α-KG or 40 mM lactate) were dissolved in reaction buffer (20 mM Tris PH = 8.0, 300 mM NaCl, 10 mM ascorbate, 0.5 mM FeSO_4_ and 1 mM DTT). Three settings were used: (1) inject 1.5 mM α-KG to 0.5 mg/ml PHD2; (2) inject 40 mM lactate to 0.5 mg/ml PHD2; (3) inject 1.5 mM α-KG to 0.5 mg/ml PHD2 + 20 mM lactate mixture. Sequential heat pulses for each injection were detected with MicroCal PEAQ-ITC Automated Ultrasensitive Isothermal Titration Calorimeter (Malvern). Data were analyzed with MicroCal PEAQ-ITC Analysis Software.

### Docking

The structure of PHD2 binding with its natural substrate α-KG (5L9B) was obtain from RCSB Protein Data Bank^52^. Docking analysis between protein and lactate was done by Hex 8.0.0 with default parameters^53^. The euclidean distance between the protein and the α-KG/lactate was calculated by in-house R script, and the amino acids with a distance of less than 5 Å from α-KG and lactate were marked.

### Total RNA isolation and real-time PCR

Total RNA was extracted by RNAiso Plus (Takara) and reverse transcribed into first strand cDNA using the RT Reagent Kit (Takara, #RR037A). Real-time PCR reactions were performed using TB Green Premix Ex Taq II (Takara, #RR820A) on a 7900 HT (Applied Biosystems), normalized with *Rps18* or *β-Actin*. The primer sequences used are listed in Supplementary Table 3.

### Western Blotting

Proteins were extracted from tissues or cells in RIPA buffer (NaCl 150 mM, Nonidet P-40 1%, sodium deoxycholate 0.5%, SDS 0.1%, Tris 25 mM, PH = 7.4) containing protease inhibitor cocktail (Merck). PVDF membrane was probed with primary antibodies at 4 °C overnight and incubated with HRP-conjugated secondary antibodies in room temperature for 1 hr. The protein bands were visualized by ECL Western Blotting Substrates (Bio-Rad). Antibodies used are: PDH2 (1:1000, Cell Signaling Technology, #4835 S, D31E11), iNOS (1:1000, Thermo Fisher Scientific, #PA3-030A), HIF-1α (1:1000, Proteintech, #20960-1-AP), HIF1α-OH-564 (1:1000, Cell Signaling Technology, #3434 S, D43B5), LDHA (1:1000, Cell Signaling Technology, #2012S), β-Actin (1:1000, Cell Signaling Technology, #4970 S, 13E5), His-tag (1:1000, R&D Systems, #MAB050R, AD1.1.10), rabbit IgG (1:2000, Cell Signaling Technology, #7074P2), mouse IgG light chain (1:2500, Cell Signaling Technology, #58802 S, D3V2A).

### RNA sequencing and data analysis

Total RNA was extracted from human omental adipose tissues using RNeasy Lipid Tissue Mini Kit (Qiagen, #74804). The quality control, RNA sequencing and data analysis were performed by Novogene Co.,Ltd. Libraries were generated using NEBNext® UltraTM RNA Library Prep Kit for Illumina (New England Biolabs, #E7530L) and paired-end clean reads were mapped to the reference genome using HISAT2 software. Differential expression analysis was performed with DESeq2 and edgeR R package. GO enrichment was performed by Metascape^54^. Heatmap was drawn with Morpheus (https://software.broadinstitute.org/morpheus). Transcription factor analysis was performed with Enrichr Submissions TF-Gene Coocurrence on Enrichr (https://maayanlab.cloud/Enrichr/enrich#) and diagrammed by Appyters (https://appyters.maayanlab.cloud/Enrichment_Analysis_Visualizer/). Cytoscape 3.7.1 was used to draw Network diagram.

#### In vitro ^13^C- lactate tracing and metabolomics analysis

Mouse inflammatory BMDM was cultured in 12 well plate with medium containing 20 mM ^13^C-C3-lactate (Sigma Aldrich #490040). 24 hrs later, the cells were washed once with cold NaCl (0.9% w/v), harvested with 400 μl of ice-cold methanol, and freezed at −80 °C for 30 min, followed by addition of 100 μl of ice-cold H2O. The cell lysates were harvested and subjected to GC/MS sample preparation and analysis (Profleader Co.). For metabolomics analysis, mouse inflammatory BMDM was treated with 20mM L-lactate or NaCl for 24 hrs. The cells were subjected to high throughput targeted quantification for metabolites by BGI company (HM350 Metabolome Kit) using liquid chromatography-tandem mass spectrometry (LC-MS/MS). Another parallel plate of the cells with the same treatment was subjected to protein concentration measurement using BCA.

#### Caspase-1 activity measurement

Caspase-1 activity was measured using Caspase-1 Colorimetric Assay Kit (R&D, #K111-100)^55^. Briefly, BMDMs were lysed in a cell lysis buffer containing 10 mM DTT. After centrifugation, lysates (10 μg) were mixed with YVAD-pNA substrate (200 µM) and incubated at 37 °C for 1 hr, followed by reading at 405 nm in a microplate reader (Biotek).

### Statistical and reproducibility

All analyses were performed with Statistical Package for Social Sciences version 14.0 (SPSS, Chicago. IL). Data were expressed as mean ± SEM unless stated elsewhere. For comparison of three or more experimental conditions, one-way or two-way analysis of variance (ANOVA) was applied for comparisons between multiple experimental groups, followed by post hoc analysis with the Bonferroni correction. Two-tailed student’s *t* test was used for comparison of two experimental conditions with normal distribution. Comparisons with *s* < 0.05 were considered statistically significant. In vitro experiments were repeated three times independently with similar results. Animal experiments were performed at least twice with each animal as a biologically independent sample.

### Reporting summary

Further information on research design is available in the Nature Research Reporting Summary linked to this article.

## Supplementary information

Supplementary Information

Reporting Summary

## Data Availability

The data supporting the findings of this study are available within the paper and its supplementary information files and Source Data file. The RNASeq data generated in this study were deposited in the GEO database with the primary accession code GSE211218 (GSM6456214 -GSM6456227). The RNASeq data of the epididymal white adipose tissue of C57BL/6J mice treated with low fat diet can be retrieved from GSE91067 (GSM2420432 - GSM2420439) in GEO database. Source data are provided with this paper.

## Source data

Source Data
