# Supplementary material for: Adipocyte-derived lactate is a signalling metabolite that potentiates adipose macrophage inflammation via targeting PHD2
Source: Nat Commun. 2022 Sep 5;13:5208. doi: 10.1038/s41467-022-32871-3 (PMC9445001; doi:10.1038/s41467-022-32871-3)
Supplement: Supplementary file 1 — Supplementary Information [file 41467_2022_32871_MOESM1_ESM.pdf]

Supplementary information

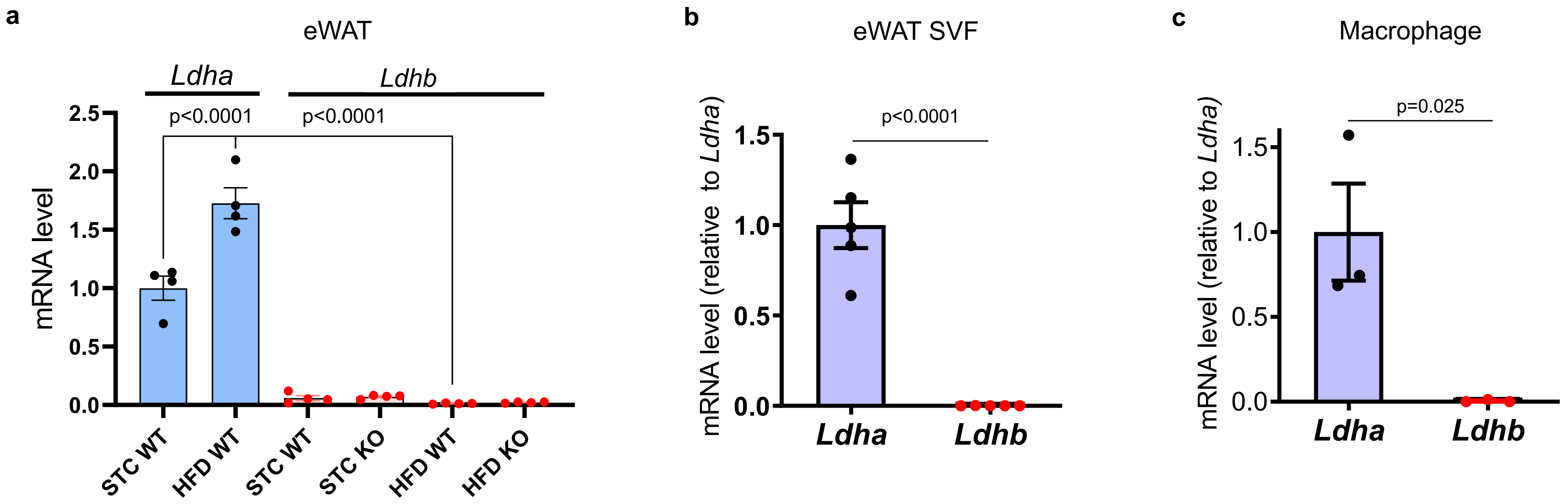

Supplementary Fig. 1 LDHA is the predominant LDH isoform in both adipose tissue and macrophage. mRNA expression of *Ldha* and *Ldhb* in **(a)** whole epididymal tissue (eWAT), (n=4 biologically independent animals), **(b)** stromal vascular fraction of eWAT (eWAT SVF), (n=5 biologically independent animals), and (**c**) bone marrow derived macrophage (n=3 biologically independent animals) was examined by real time PCR. Data represent mean ± SEM; Significance was calculated using one-way ANOVA with post hoc Bonferroni correction **(a)**, or two-tailed student's t test **(b, c)**.

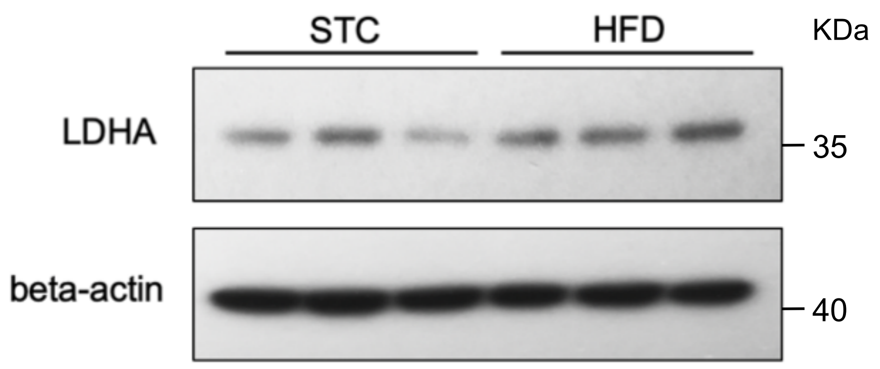

Supplementary Fig. 2 LDHA is not significantly elevated in subcutaneous white adipose tissue (scWAT) after high fat diet. 8-week-old male C57BL/6J mice were fed with standard chow (STC) or high fat diet (HFD) for 3 months. LDHA protein level in mouse scWAT was determined by Western blotting.

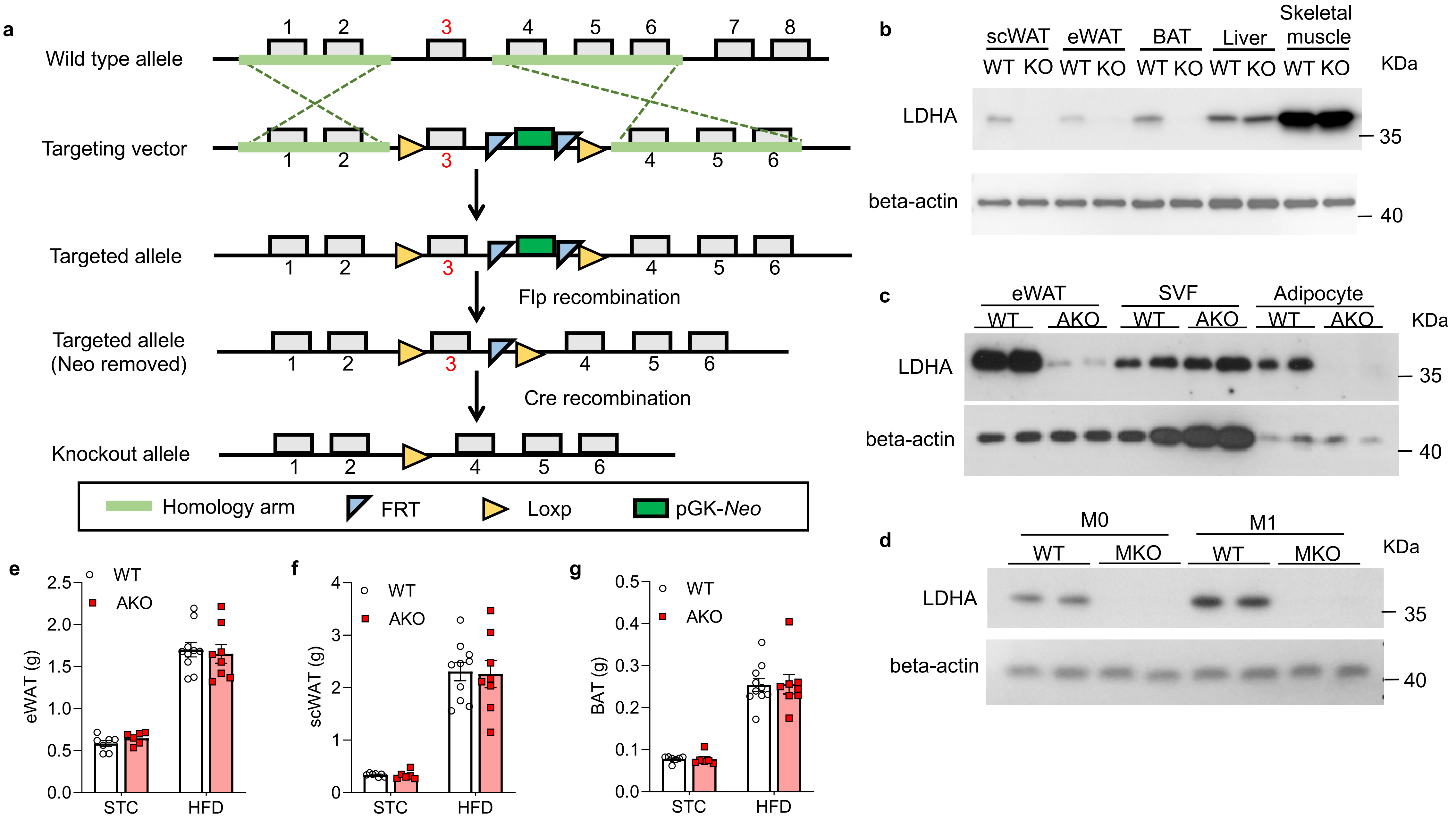

Supplementary Fig. 3 Generation of Adipocyte and myeloid cell specific *Ldha* knockout mice. a Strategy of cell type-specific *Ldha* knockout mice. The 3rd exon of mouse *Ldha* gene is flanked by loxP sites. The selection marker pGK-*Neo* gene was deleted by crossing the F1 mice with *Flp*+ mice. Flox positive homozygous mice were then used to mate with Cre+ mice to generate adipocyte (AKO) and myeloid cell (MKO)-specific *Ldha* KO mice. **b** Western blotting of LDHA in different tissues of male AKO mice. WT mice without Cre transgene was used as the control. **c** eWAT of male WT and AKO mice were fractionated to stromal vascular fraction (SVF) or mature adipocytes and LDHA level in each fraction was determined by Western blotting; n=2 biologically independent animals. **d** Western blotting of LDHA in bone marrow derived M0 and M1 macrophages of WT and MKO mice ; n=2 biologically independent samples. (**e**-**g**) Weights of (**e**) epididymal white adipose tissue (eWAT), (**f**) subcutaneous white adipose tissue (scWAT) and **(g)** brown adipose tissue (BAT), STC-WT: n=7; STC-AKO: n=6; HFD-WT: n=10; HFD-AKO: n=8 biologically independent animals. Data represent mean ± SEM; Significance was calculated using two way ANOVA with post hoc Bonferroni correction (e-g).

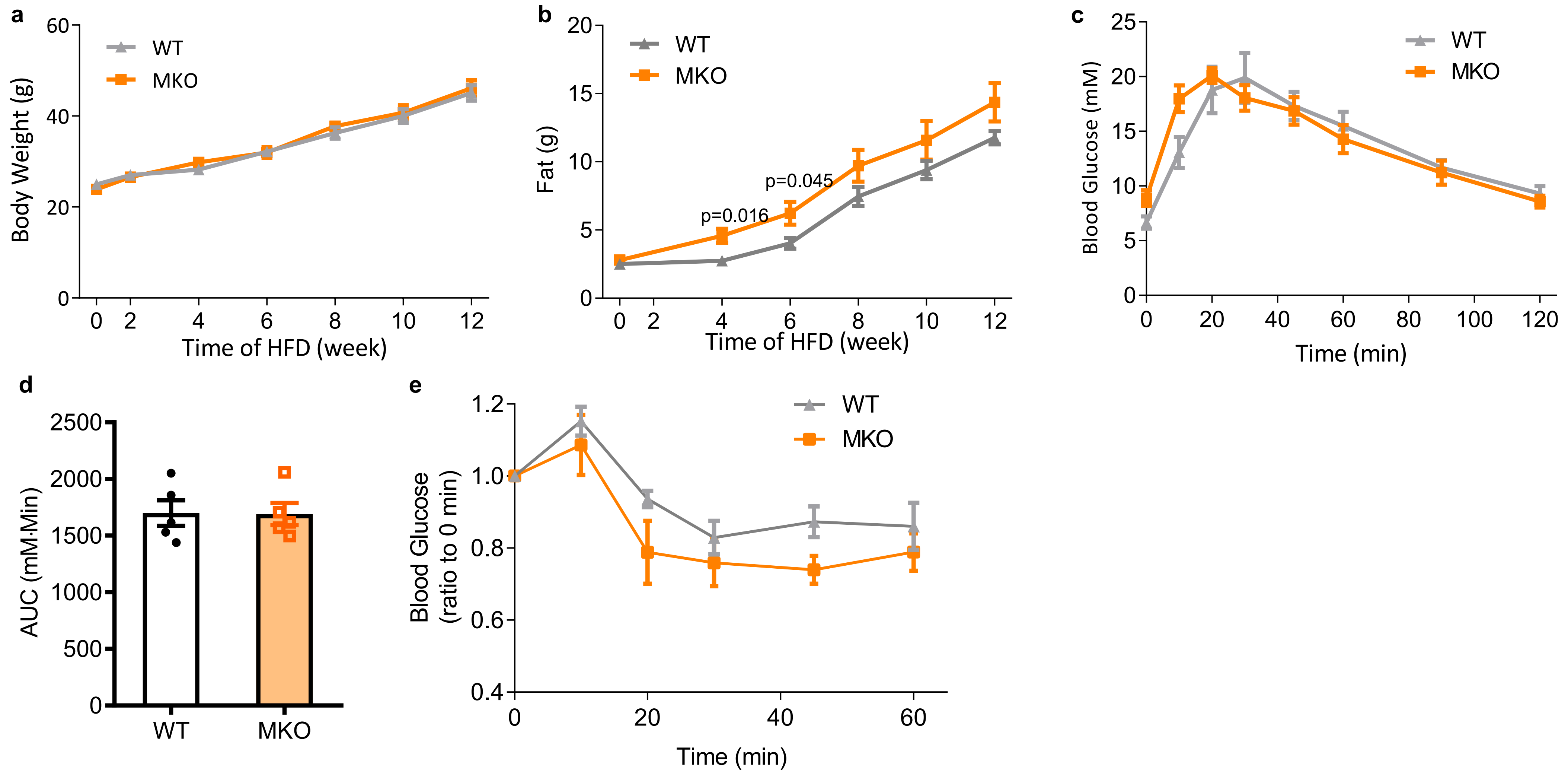

Supplementary Fig. 4 Myeloid cell-specific deletion of *Ldha* does not cause significant changes in glucose metabolism in obese mice. Wildtype (WT) and myeloid cell-specific *Ldha* KO (MKO) mice were fed with high fat diet (HFD). **a** Body weight of the mice. **b** Fat mass of the mice. **c** Glucose tolerance test (GTT) and **d** Area under the curve (AUC) of the GTT results. **e** Insulin tolerance test (ITT). n = 5 biologically independent animals in all panels; Data represent mean ± SEM; Significance was calculated using one way ANOVA with post hoc Bonferroni correction **(a, b, c, e)**, or two-tailed student's t test **(d)**.

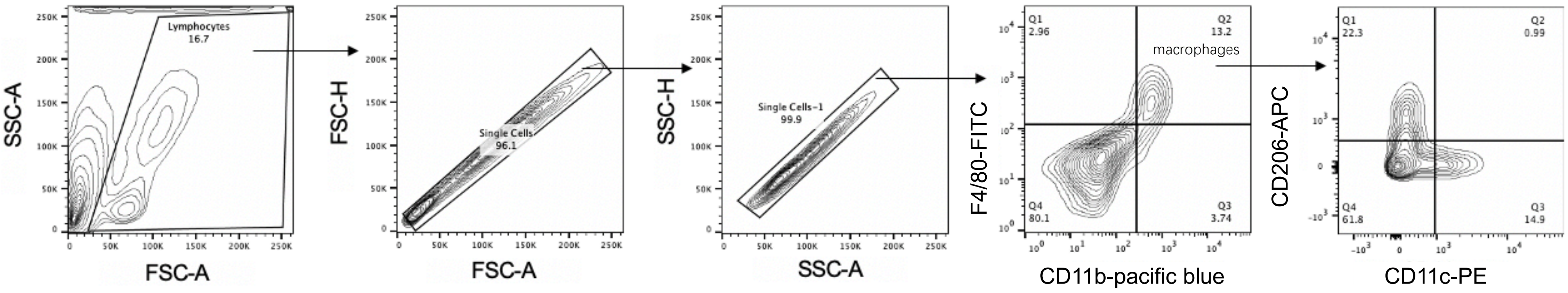

Supplementary Fig. 5 Gating strategy of flow cytometry for analysis of the adipose tissue macrophage number and subtypes.

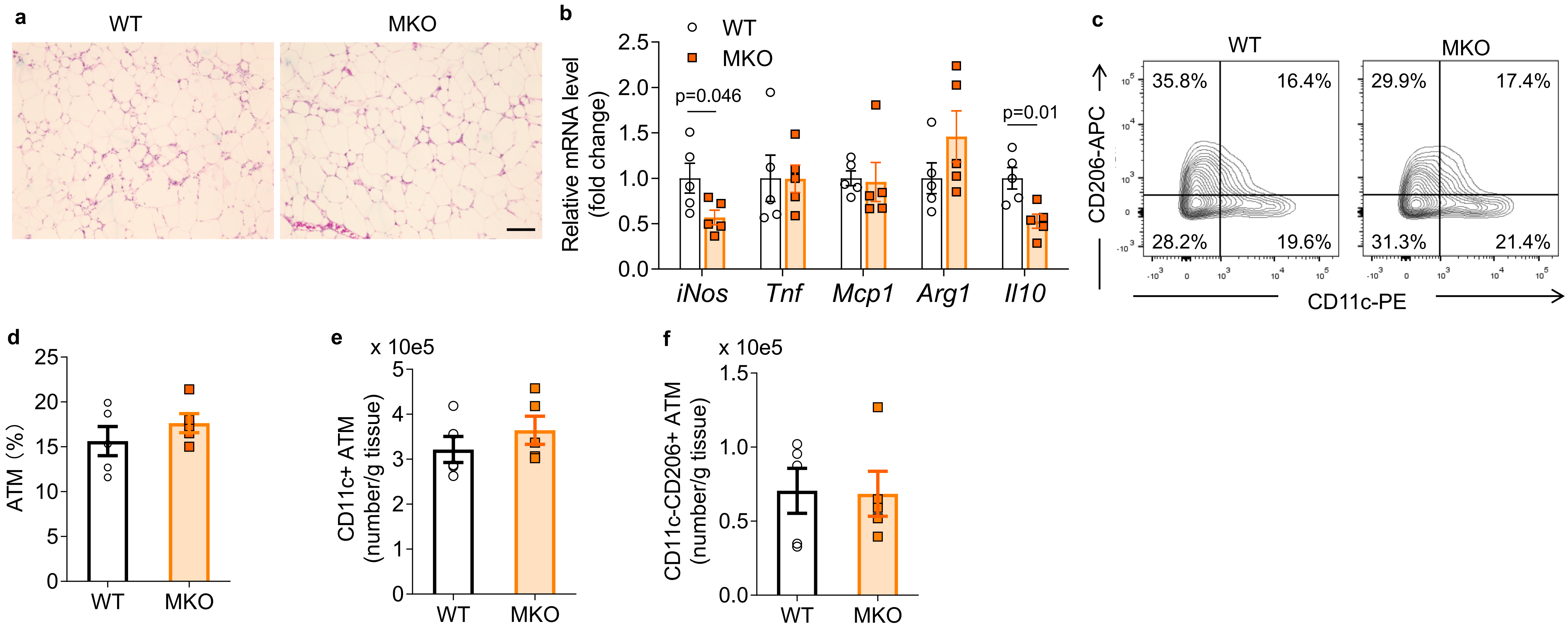

Supplementary Fig. 6 Obese WT and MKO have similar level of adipose inflammation. Wildtype (WT) and myeloid cell-specific *Ldha* KO (MKO) mice were fed with high fat diet (HFD) for 12 weeks before epididymal white adipose tissue (eWAT) was isolated for analysis. **a** HE staining of eWAT. Scale bar, 20μm. **b** mRNA level of inflammatory cytokines in eWAT. n=5 biologically independent animals. (**c**-**f**) Stromal vascular fraction (SVF) in eWAT was subjected to flowcytometry analysis for macrophage subtypes. **c** Representative flow chart for CD11c and CD206 staining gated with F4/80+ cells in SVF. **d** Percentage of adipose tissue macrophages (ATM, F4/80+) in total SVF. n=5 biologically independent animals. (**e**-**f**) Number of different subtypes of ATMs in adipose. n=5 biologically independent animals. Data represent mean ± SEM; n = 5 biologically independent animals; Significance was calculated using two way ANOVA with post hoc Bonferroni correction **(b)**, or two-tailed student's t test **(d, e, f)**.

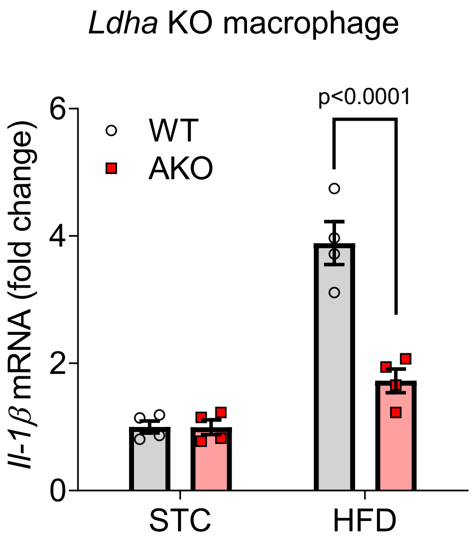

Supplementary Fig. 7 Adipocyte-derived lactate influences *Il-1β* expression in macrophage. Wildtype (WT) and adipocyte-specific *Ldha* KO (AKO) mice were fed with standard chow (STC) or high fat diet (HFD) for 3 months, after which the conditioned medium of epididymal white adipose tissue (eWAT) was collected and cocultured with bone marrow derived macrophages isolated from 8-week old lean myeloid cell-*Ldha* KO (MKO) mice. mRNA expression of *Il-1β* in MKO macrophages was determined by real time PCR. Data represent mean ± SEM; n=4 biologically independent MKO mice. Significance was calculated using two way ANOVA with post hoc Bonferroni correction.

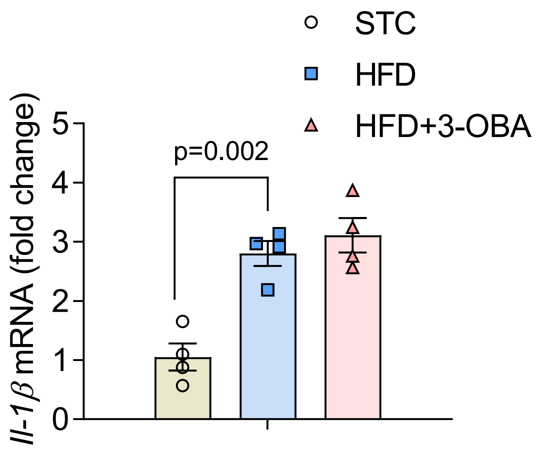

Supplementary Fig. 8 Inhibition of GPR81 does not affect the effect of the obese eWAT CM on *Il-1β* expression. Conditioned medium (CM) of epididymal white adipose tissue (eWAT) was collected from WT or AKO mice fed with standard chow (STC) or high fat diet (HFD). Bone marrow derived macrophages were cocultured in CM for 24 hr with or without 3-OBA (100 μM). *Il-1β* mRNA expression was examined by real time PCR. Data represent mean ± SEM; n=4 biologically independent samples; Significance was calculated using one way ANOVA with post hoc Bonferroni correction.

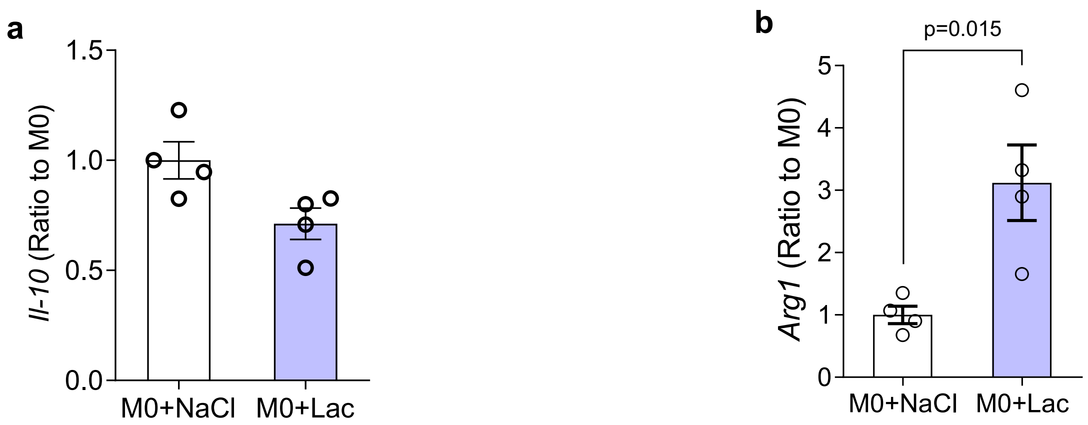

Supplementary Fig. 9 The effects of lactate on unelicited macrophage. Unelicited bone marrow derived macrophages (M0) were treated with 20mM lactate or 20mM NaCl as control for 24hr. M2 related genes (**a**) *Il-10* and (**b**) *Arg1* were determined by real time PCR. n=4 biologically independent samples. Data represent mean ± SEM; Significance was calculated using two-tailed student's t test.

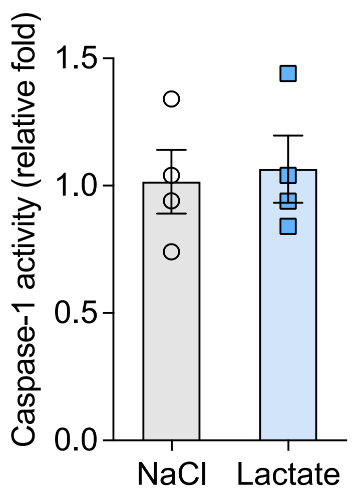

Supplementary Fig. 10 Caspase-1 activity was unaltered in pro-inflammatory macrophages by lactate. Caspase-1 activity was measured in pro-inflammatory mouse bone marrow derived macrophages in the absence or presence of lactate (20mM) for 24 hr. n=3 biologically independent samples. Data represent mean ± SEM; Significance was calculated using two-tailed student's t test.

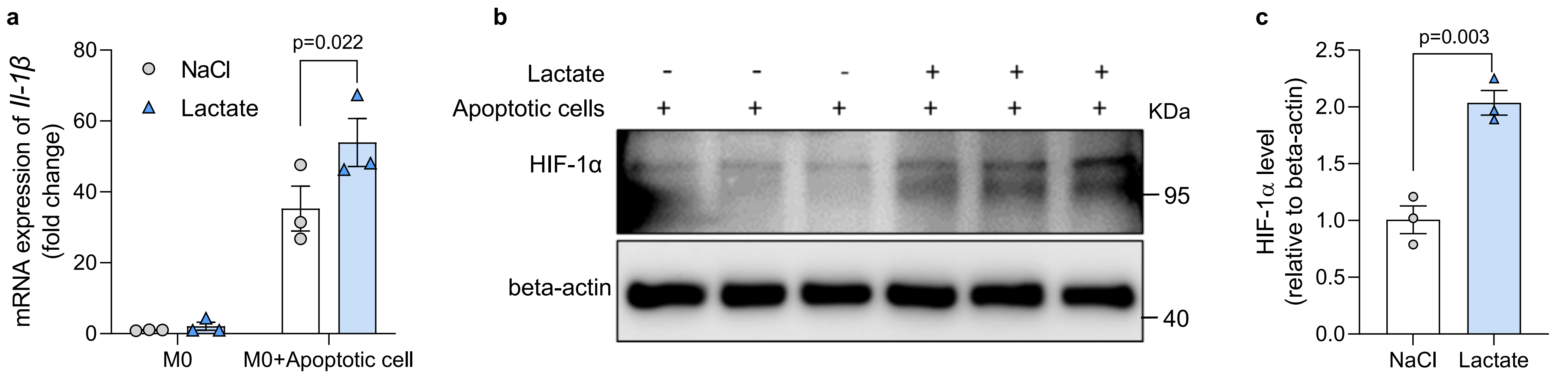

Supplementary Fig. 11 Lactate induces macrophage inflammation and HIF1α in the presence of apoptotic cells. Unelicited bone marrow derived macrophages (M0) were incubated with apoptotic cells and treated with 20mM lactate or 20mM NaCl as control for 24 hr. **a** mRNA expression of *Il-1β* was determined by real time PCR. n=3 biologically independent samples. **b** Western blotting of HIF1α. **c** Quantification of Western blot result. n=5 biologically independent samples. Data represent mean ± SEM; Significance was calculated using two way ANOVA with post hoc Bonferroni correction (a) and two-tailed student's t test (c).

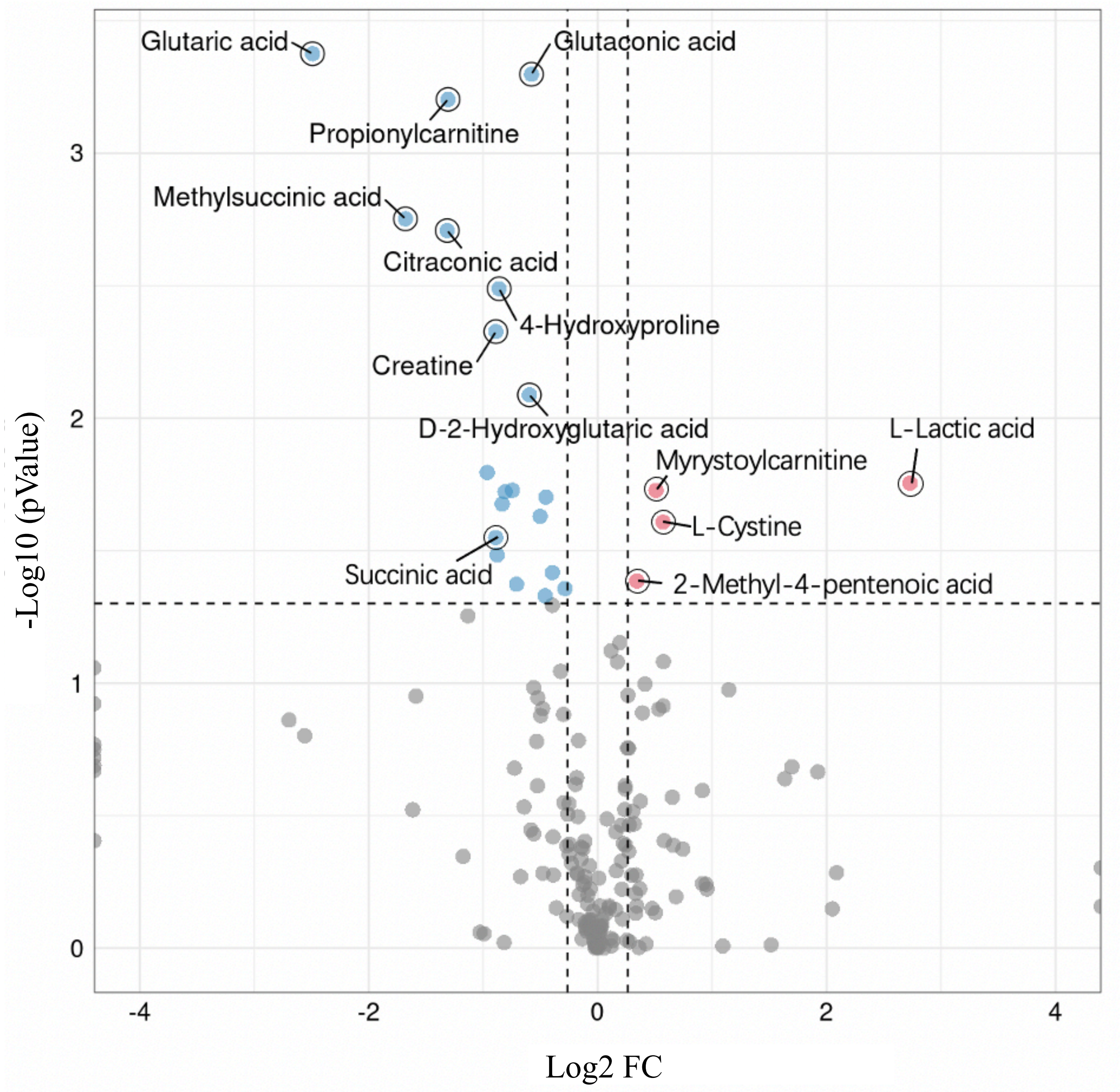

Supplementary Fig. 12 Exogenous lactate treatment does not significantly alter glycolysis and TCA cycle reaction in pro-inflammatory macrophage. Mouse inflammatory bone marrow-derived macrophage was treated with 20mM L-lactate or NaCl for 24 hr and the cell lysate was subjected to high throughput targeted quantification for metabolites using liquid chromatography-tandem mass spectrometry (LC-MS/MS). Volcano diagram of the metabolites (Lactate vs. NaCl). Top changed metabolites and Succinic acid are illustrated.

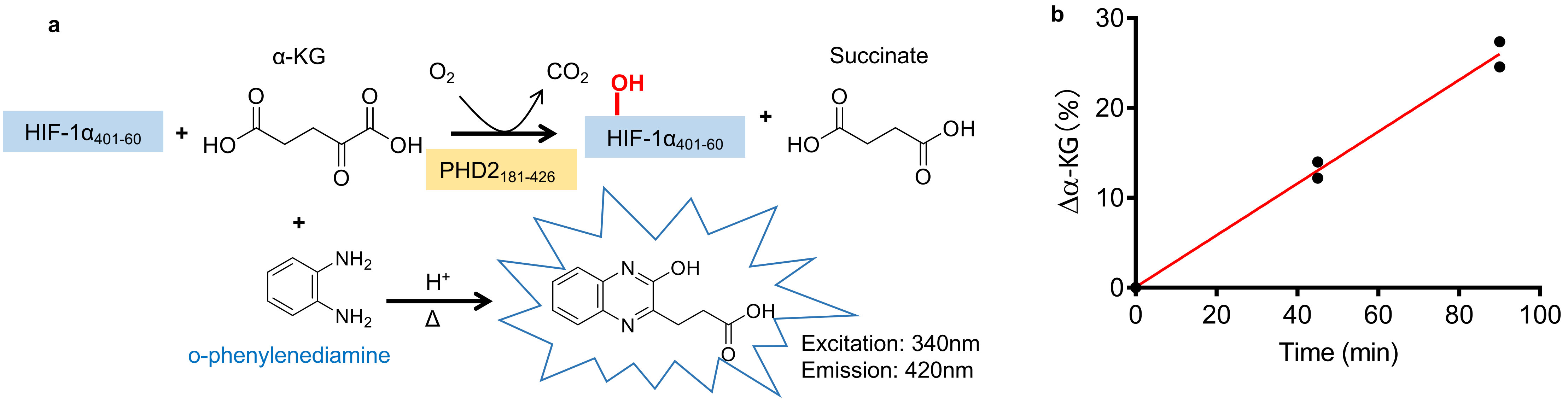

Supplementary Fig. 13 Establishment of PHD2 *in vitro* activity assay. a Diagram of the fluorometric *in vitro* assay for measurement of PHD2 activity. PHD2 catalyzes the hydroxylation of HIF-1α during which α-KG is decarboxylated to succinate. α-KG reacts with o-phenylenediamine to generate a fluorescent product. Thus the reduction in α-KG in the reaction is measured indirectly by detecting the fluorescence intensity at Ex 340 nm/Em 420 nm, which indicates the activity of PHD2. **b** Validation of the *in vitro* assay. The decremence in α-KG (Δα-KG) increased in a time dependent manner.

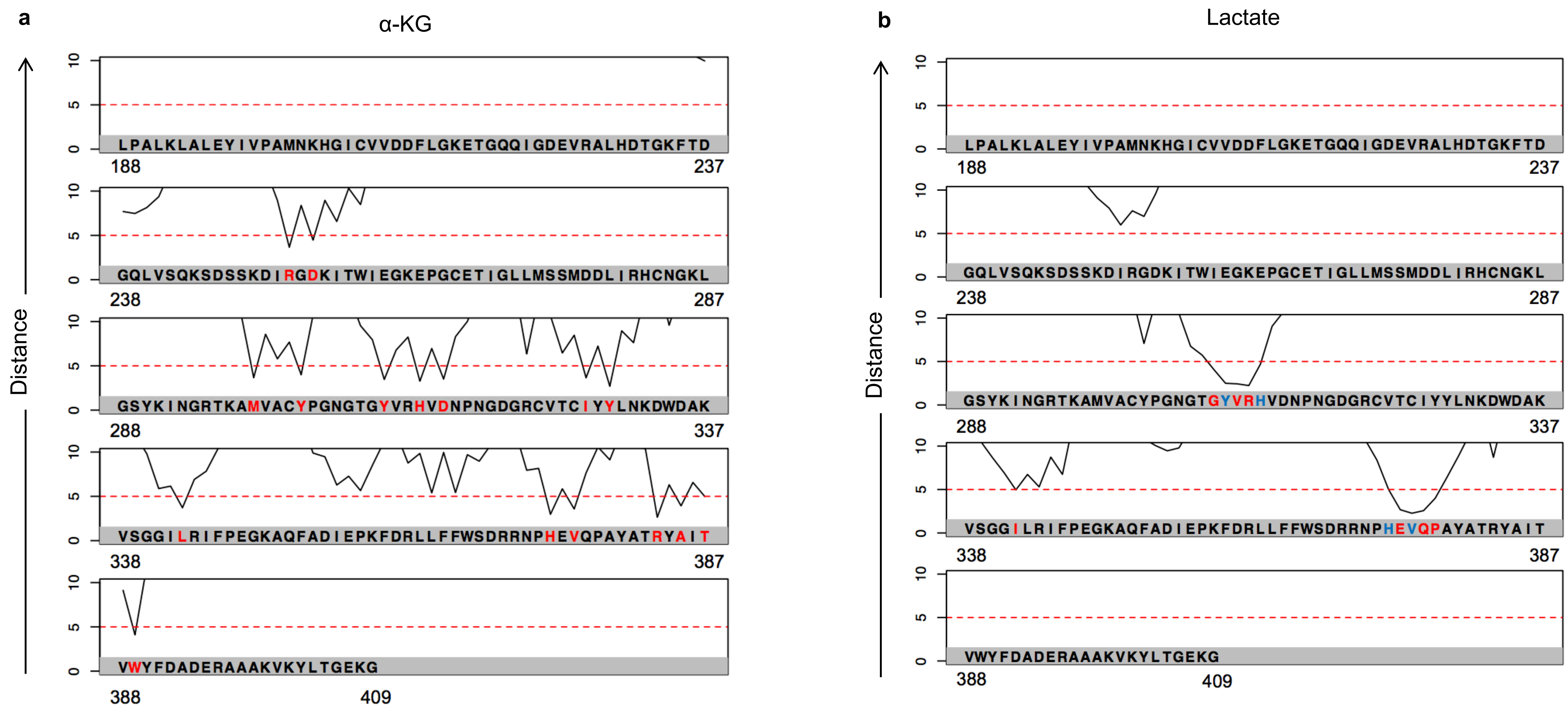

Supplementary Fig. 14 Lactate interferes with the binding between α-KG and PHD2. a Distance of amino acid residues of PHD2 to α-KG. Amino acids with distance < 5Å are highlighted in red. **b** Distance of amino acid residues of PHD2 to lactate. Amino acids with distance < 5Å are highlighted in red or blue. The blue ones (Arg252, Asp254, Tyr310, Val376) are those also involved in binding with α-KG.

Variables	Correlation analysis (n=65)	High_Lac (n=7)	Low_Lac (n=7)	*p* value (High_Lac *vs.* Low_Lac)
Age	40.54±14.61	36.71±9.91	36.57±10.90	0.88
Sex (M/F)	26/39	4/3	2/5	0.28
Weight (kg)	86.75±28.53	106.36±16.18	111.3±25.06	0.73
height (cm)	165.66±9.75	172.83±5.73	170.15±5.81	0.52
BMI (kg/m2)	32.44±11.26	36.33±4.93	42.46±7.72	0.13
Lactate in omental fat (mMol/g)	15.02±10.65	25.52±8.11	6.39±0.61	9.00E-05
Fasting glucose (mM)	5.57±1.62	7.88±1.75	5.54±0.92	0.01
Fasting insulin (μU/mL)	15.79±12.16	25.88±13.22	21.08±9.25	0.48
HOMA-IR	3.84±3.85	8.61±3.8	5.36±2.71	0.114
HOMA-beta	172.06±131.47	149.73±116.82	222.21±119.45	0.309
SBP (mm Hg)	122.98±13.55	128.17±4.84	134.17±13.36	0.367
DBP (mm Hg)	76.88±11.87	90.17±7.88	87.67±11.69	0.700
Waist circumference (cm)	122.58±15.17	117.53±10.92	125.76±18.97	0.375

Supplementary Table 1. Anthropometric parameters of the human subjects. BMI, body mass index; SBP, systolic blood pressure; DBP, diastolic blood pressure. Data were shown as means ± SD. Difference was determined by two-tailed Student's t test.

Name	p value
SPI1	2.13E-89
PLEK	1.09E-67
TFEC	1.18E-65
IRF5	1.21E-64
LYL1	1.21E-63
RNASE2	1.20E-62
IKZF1	9.94E-59
IRF8	5.67E-54
BATF	8.07E-46
SP140	7.55E-40
IGHM	2.16E-37
SPIC	2.16E-37
AKNA	1.95E-33
POU2F2	6.65E-32
CEBPE	1.17E-29
ZNF705A	6.32E-29
SEMA4A	1.11E-24
ZBP1	1.11E-24
RUNX3	5.32E-24
SPIB	2.51E-23
IKZF3	2.51E-23
NR1H3	1.16E-22
VENTX	2.35E-21
BATF3	2.35E-21
RNF166	1.03E-20
FLI1	4.42E-20
NFE2	4.42E-20
PLXNC1	1.87E-19
IRF7	3.13E-18
ELF4	1.25E-17
SP110	1.25E-17
IRF4	4.88E-17
CD36	7.02E-16
CEBPA	7.02E-16
MAFB	9.31E-15
STAT4	3.29E-14
TBX21	3.29E-14
FOXP3	1.14E-13
ZNF683	3.86E-13
HHEX	1.28E-12
SP100	4.17E-12
TFEB	4.17E-12
ZDHHC19	1.33E-11
NR1I3	1.25E-10
PARP12	3.73E-10
IFI16	1.09E-09
GFI1	1.09E-09
STAT1	1.09E-09
STAT5A	1.09E-09
SNAI3	1.09E-09
SSH2	1.09E-09
ZNF385A	3.09E-09
STAT6	3.09E-09
CEBPB	3.09E-09
CREB3L3	3.09E-09
ZNF438	8.58E-09
IRF9	8.58E-09
PLXNB2	2.33E-08
HLX	2.33E-08
ZNF831	2.33E-08
BATF2	2.33E-08

Name	p value
NR1H4	6.17E-08
TAL1	1.60E-07
ZNF366	4.03E-07
IRF1	4.03E-07
HNF4A	4.03E-07
TRAFD1	4.03E-07
MXD1	9.91E-07
SCML4	9.91E-07
ZFP36	9.91E-07
ZNF746	9.91E-07
STAT2	9.91E-07
RELB	9.91E-07
ZBTB32	2.38E-06
VDR	2.38E-06
JUNB	5.56E-06
SLC22A4	5.56E-06
RUNX1	5.56E-06
NFKB2	5.56E-06
KLF2	5.56E-06
ZEB2	5.56E-06
ARID3A	1.27E-05
TSC22D3	1.27E-05
HIF-1A	1.27E-05
TSC22D4	1.27E-05
ZBED1	2.81E-05
GFI1B	2.81E-05
CARHSP1	2.81E-05
NFE2L2	2.81E-05
ZNF101	2.81E-05
GATA1	6.08E-05
MBD2	6.08E-05
PAX5	6.08E-05
ASCL2	0.000128
NR0B2	0.000128
ZNFX1	0.000128
ZFP36L2	0.000128
NR1I2	0.000128
ZNF217	0.000128
CREB3	0.000128
FOXN2	0.000128
MEF2B	0.000262
ZMAT3	0.000262
AIRE	0.000262
MAF	0.000262
ZNF679	0.000262
KLF1	0.000262
TCF7	0.000262
ZC3HAV1	0.000262
NR2F6	0.000262
NFIL3	0.000262
FOS	0.000262
BACH1	0.000262
MXD4	0.000262
PLEK2	0.000521
PLXND1	0.000521
RUNX2	0.000521
BCL6	0.000521
ZFYVE26	0.000521
TGIF1	0.000521
JDP2	0.000521
HMG20B	0.000521

Name	p value
HOPX	0.000521
ID2	0.000521
ATF6	0.000521
IRF3	0.000521
ATF3	0.000521
RXRA	0.001007
ZNF267	0.001007
ATF5	0.001007
ZNF281	0.001007
RNF125	0.001007
MLX	0.001007
MSC	0.001007
ZNF586	0.001007
H1FOO	0.001007
SSH3	0.001892
STAT3	0.001892
PRDM1	0.001892
IRF2	0.001892
HSF5	0.001892
EOMES	0.001892
ZCCHC6	0.001892
ZNF467	0.001892
MST1R	0.001892
FOSL2	0.001892
REL	0.001892
ZNF277	0.001892
ZNF200	0.001892
MAFK	0.003452
ZNF296	0.003452
PBX4	0.003452
ZNF80	0.003452
RPA4	0.003452
TCF23	0.003452
TIGD6	0.003452
ELF3	0.003452
NCOA3	0.003452
GATA3	0.003452
ZBTB43	0.003452
ARNTL2	0.003452
RCOR1	0.003452
ZNF317	0.003452
RAG1	0.003452
NR3C1	0.006112
ZNF524	0.006112
MYC	0.006112
TBX10	0.006112
NFKB1	0.006112
EGR2	0.006112
TBX19	0.006112
MITF	0.006112
EHF	0.006112
TP53	0.006112
HNF1A	0.006112
HIST1H1T	0.006112
PPARG	0.006112
USF1	0.006112
ZNF341	0.006112
ARID5B	0.006112
TIGD2	0.006112
RFXANK	0.006112
CTCFL	0.006112

Supplementary Table 2. Enrichr Submissions TF-Gene Coocurrence analysis for transcription factors enriched in high lactate adipose tissues. All the transcription factors with p value <0.01 are listed.

Name	Forward (5' – 3')	Reverse (5' – 3')	Species	Note
Arg1	TGGCTTGCGAGACGTAGAC	GCTCAGGTGAATCGGCCTTTT	Mouse	Q-PCR
F4/80	CTTTGGCTATGGGCTTCCAGTC	GCAAGGAGGACAGAGTTTATCGTG	Mouse	Q-PCR
Gapdh	TGAAGCAGGCATCTGAGGG	CGAAGGTGGAAGAGTGGGAG	Mouse	Q-PCR
Hif-1a	ACCTTCATCGGAAACTCCAAAG	CTGTTAGGCTGGGAAAAGTTAGG	Mouse	Q-PCR
IL-1b	GGGCCTCAAGGAAAAGAATC	TTCTGCTTGAGAGGTGCTGA	Human	Q-PCR
iNOS	CCTGAGCTCTTCGAAATCCCA	CCCGAAACCACTCGTATTTGG	Human	Q-PCR
MCP1	TCGCTCAGCCAGATGCAATCAA	TTTGCTTGTCCAGGTGGTCC	Human	Q-PCR
TNF	ACTTTGGAGTGATCGGCC	GCTTGAGGGTTTGCTACAAC	Human	Q-PCR
Il-10	GCTCTTACTGACTGGCATGAG	CGCAGCTCTAGGAGCATGTG	Mouse	Q-PCR
Il-1b	GCAACTGTTCCTGAACTCAACT	ATCTTTTGGGGTCCGTCAACT	Mouse	Q-PCR
iNos	CCAAGCCCTCACCTACTTCC	CTCTGAGGGCTGACACAAGG	Mouse	Q-PCR
Ldhb	TGCGTCCGTTGCAGATGAT	TTTCGGAGTCTGGAGGAACAA	Mouse	Q-PCR
Ldha	CAAAGACTACTGTGTAACTGCGA	TGGACTGTACTTGACAATGTTGG	Mouse	Q-PCR
Mcp1	CATCCACGTGTTGGCTCA	GATCATCTTGCTGGTGAATGA	Mouse	Q-PCR
Phd2	TAAACGGCCGAACGAAAGC	GGGTTATCAACGTGACGGACA	Mouse	Q-PCR
Rps18	AGTTCCAGCACATTTTGCGAG	TCATCCTCCGTGAGTTCTCCA	Mouse	Q-PCR
Tnf	ACGGCATGGATCTCAAAGAC	AGATAGCAAATCGGCTGACG	Mouse	Q-PCR
Vegf	GGAGATCCTTCGAGGAGCACTT	GGCGATTTAGCAGCAGATATAAGAA	Mouse	Q-PCR
Glut1	TCAACACGGCCTTCACTG	CACGATGCTCAGATAGGACATC	Mouse	Q-PCR
Cre R	AGCGATGGATTTCCGTCTCTGG	AGCTTGCATGATCTCCGGTATTGAA	Mouse	Genotyping
Ldha Flox	TCACACCTAGCTTTGCAGAATACT	TCAGGCCCGAGGAGGAACTTA	Mouse	Genotyping

Supplementary Table 3. Sequence of the primers used in this study.
