# Supplementary material for: Adipocyte-derived lactate is a signalling metabolite that potentiates adipose macrophage inflammation via targeting PHD2
Source: Nat Commun. 2022 Sep 5;13:5208. doi: 10.1038/s41467-022-32871-3 (PMC9445001; doi:10.1038/s41467-022-32871-3)
Supplement: Supplementary file 2 — Reporting Summary [file 41467_2022_32871_MOESM2_ESM.pdf]

nature research

Corresponding author(s): Aimin Xu &Xiaoyan Hui

Last updated by author(s): Jul 3, 2022

Reporting Summary

Nature Research wishes to improve the reproducibility of the work that we publish. This form provides structure for consistency and transparency in reporting. For further information on Nature Research policies, see our Editorial Policies and the Editorial Policy Checklist.

Statistics

For all statistical analyses, confirm that the following items are present in the figure legend, table legend, main text, or Methods section.

n/a	Confirmed	
☐	☒	The exact sample size (*n*) for each experimental group/condition, given as a discrete number and unit of measurement
☐	☒	A statement on whether measurements were taken from distinct samples or whether the same sample was measured repeatedly
☐	☒	The statistical test(s) used AND whether they are one- or two-sided<br>*Only common tests should be described solely by name; describe more complex techniques in the Methods section.*
☐	☒	A description of all covariates tested
☐	☒	A description of any assumptions or corrections, such as tests of normality and adjustment for multiple comparisons
☐	☒	A full description of the statistical parameters including central tendency (e.g. means) or other basic estimates (e.g. regression coefficient) AND variation (e.g. standard deviation) or associated estimates of uncertainty (e.g. confidence intervals)
☐	☒	For null hypothesis testing, the test statistic (e.g. *F*, *t*, *r*) with confidence intervals, effect sizes, degrees of freedom and *P* value noted<br>*Give P values as exact values whenever suitable.*
☒	☐	For Bayesian analysis, information on the choice of priors and Markov chain Monte Carlo settings
☒	☐	For hierarchical and complex designs, identification of the appropriate level for tests and full reporting of outcomes
☐	☒	Estimates of effect sizes (e.g. Cohen's *d*, Pearson's *r*), indicating how they were calculated

Our web collection on statistics for biologists contains articles on many of the points above.

Software and code

Policy information about availability of computer code

Data collection	In the RNA-seq experiment, the paired-end clean reads were mapped to the reference genome using HISAT2 software.<br>The flow cytometry data were collected with BD FACSDiva (version 9.0).<br>The Real-time PCR data were collected with StepOne Software (version 2.2.2).<br>The immunofluorescence staining images were collected with ZEN (version 2.3, BLUE Edition).<br>The lactate assay data, LDH activity assay data, ELISA data and PHD2 activity assay data were collected with CLARIOstar Touch software (version 5.01R2).
Data analysis	Differential expression analysis for RNA-seq data was performed with DESeq2 (version 1.30.1) and edgeR (version 3.32.1) packages in R (version 4.0.4).<br>GO enrichment was performed by Metascape (https://metascape.org/gp/index.html).<br>Heatmap was drawn with Morpheus (https://software.broadinstitute.org/morpheus).<br>Transcription factor analysis was performed with Enrichr Submissions TF-Gene Coocurrence on Enrichr (https://maayanlab.cloud/Enrichr/enrich#) and diagrammed by Appyters (https://appyters.maayanlab.cloud/Enrichment_Analysis_Visualizer/).<br>Network diagram was drawn with Cytoscape (version 3.7.1).<br>Other statistical graphs were drawn with GraphPad Prism (version 8.0.2).<br>The docking of lactate and α-KG with PHD2 was performed with Hex (version 8.0.0) and visualized with Pymol (version 2.5.0).<br>The flow cytometry data were analyzed with FlowJo X (version 10.0.7r2).<br>The quantification of protein level in western blotting was performed with ImageJ (version 1.51k).<br>The isothermal titration calorimetry data were analyzed with MicroCal PEAQ-ITC Analysis Software (version 1.0.0.1259).<br>Other data analysis was performed with Statistical Package for Social Sciences (version 14.0).

For manuscripts utilizing custom algorithms or software that are central to the research but not yet described in published literature, software must be made available to editors and reviewers. We strongly encourage code deposition in a community repository (e.g. GitHub). See the Nature Research guidelines for submitting code & software for further information.

Data

Policy information about availability of data

All manuscripts must include a data availability statement. This statement should provide the following information, where applicable:

- Accession codes, unique identifiers, or web links for publicly available datasets
- A list of figures that have associated raw data
- A description of any restrictions on data availability

The authors declare that all the data supporting the findings of this study are available within the paper and its supplementary information files and Source Data file. The RNASeq data generated in this study in Figure 7k-m have been deposited were deposited in CNCB-NGDC (https://ngdc.cncb.ac.cn/omix/) with the data identifier PRJCA009360. Source data are provided with this paper.

Field-specific reporting

Please select the one below that is the best fit for your research. If you are not sure, read the appropriate sections before making your selection.

☒ Life sciences ☐ Behavioural & social sciences ☐ Ecological, evolutionary & environmental sciences

For a reference copy of the document with all sections, see nature.com/documents/nr-reporting-summary-flat.pdf

Life sciences study design

All studies must disclose on these points even when the disclosure is negative.

Sample size	The N number for all experiments, including animal experiments, in vitro experiments and clinical sample studies were listed in the figure legends or in Table 1. The sample sizes were determined based on the previous papers with similar experiments (Pan et al. J Clin Invest. 2019;129(2):834–849, DOI: 10.1172/JCI123069; Hui et al. EMBO Reports. 2017; 18:645-657, DOI: 10.15252/embr.201643184 and etc). The N number for clinical experiments was also limited by the availability of the samples that can be obtained and are enough to avoid significant interference of individual variation.
Data exclusions	No data were excluded for analysis.
Replication	All animal experiments were repeated at least twice and in vitro experiments were repeated at least three times. All results are reproducible and representative data were showed in the figures or supplementary files.
Randomization	All the allocation was preformed randomly. Mice of the same genotype and sex with similar date of birth (within one week) were randomly allocated into different treatment groups. For in vitro studies, all groups of control and treatment were allocated randomly. We strictly followed the standard laboratory operation procedures to keep the experimental environments and facilities consistent and under the same conditions. Patient samples for RNA Seq analysis were randomly picked from each group.
Blinding	Investigators were blinded to group allocation during data collection, except for some samples which require sample loading in appropriate orders, such as western blot. For measuring the lactate levels, the investigator was blinded to the other informations of the subjects. For data analysis, the investigators were divided into two groups. One group organized the data and the other group was blinded to the grouping information and performed the image quantification and data analysis.

Reporting for specific materials, systems and methods

We require information from authors about some types of materials, experimental systems and methods used in many studies. Here, indicate whether each material, system or method listed is relevant to your study. If you are not sure if a list item applies to your research, read the appropriate section before selecting a response.

Materials & experimental systems

n/a	Involved in the study	
☐	☒	Antibodies
☒	☐	Eukaryotic cell lines
☒	☐	Palaeontology and archaeology
☐	☒	Animals and other organisms
☐	☒	Human research participants
☒	☐	Clinical data
☒	☐	Dual use research of concern

Methods

n/a	Involved in the study	
☒	☐	ChIP-seq
☐	☒	Flow cytometry
☒	☐	MRI-based neuroimaging

Antibodies

Antibodies used	F4/80 (Thermo Fisher Scientific, #14-4801-85), iNOS (Thermo Fisher Scientific, #PA3-030A), HIF-1α (Proteintech, #20960-1-AP),

	HIF1α-OH-564 (Cell Signaling Technology, #3434S), PDH2 (Cell Signaling Technology, #4835S), LDHA (Cell Signaling Technology, #2012S), β-Actin (Cell Signaling Technology, #4970S,), His-tag (R&D, #MAB050R), Goat anti-Rabbit IgG (Cell Signaling Technology, #7074P2), Rabbit anti-Mouse IgG light chain (Cell Signaling Technology, #58802S), CD14 (Biolegend, #325604), Chicken anti-Rat IgG (Thermo Fisher Scientific, #A21470), Goat anti-Rabbit IgG (Thermo Fisher Scientific, #A11011), F4/80-FITC (Biolegend, #123108), CD11b-pacific blue (Biolegend, #101224), CD206-APC (Biolegend, #141708) and CD11c-PE (Biolegend, #117308).
Validation	The antibodies for iNOS, HIF-1α were validated for the IF staining for both human and mouse samples on the websites of the companies. CD14 were validated for the IF staining for human and F4/80 was validated for mouse samples IF staining.<br>F4/80-FITC, CD11b-pacific blue, CD206-APC and CD11c-PE antibodies were validated for mice cells flow cytometry studies by BioLegend.<br>iNOS, HIF-1α, PDH2, HIF1α-OH-564, LDHA, β-Actin were validated for the western blotting of mice samples on the websites of the companies.

Animals and other organisms

Policy information about studies involving animals; ARRIVE guidelines recommended for reporting animal research

Laboratory animals	Ldhafl/fl mice at the C57BL/6J background were generated by Shanghai Model Organisms Center, Inc. The mice were mated with AdipoQ-Cre and Lysozyme2-Cre transgenic mice (Jaxson Laboratory) to obtain the adipocyte and myeloid cell specific Ldha KO mice. 8-week-old male mice were used for the treatment. The mice were housed in a controlled environment (22℃ ± 1℃ and 60%–70% humidity with 12 hr light/dark cycle) and fed ad libitum with standard chow (LabDiet, #5053) or 45 kcal% high fat diet (Research Diet, #D12451)
Wild animals	The study did not involve any wild animal.
Field-collected samples	No field collected samples were used in the study.
Ethics oversight	All procedures of animals were in accordance with the research ethics guidelines for the use of laboratory animals of the Committee on the Use of Live Animals in Teaching and Research in the University of Hong Kong (CULATR No. 3967-16) and Animal Experimentation Ethics Committee in the Chinese University of Hong Kong (AEEC No. 21-051-MIS).

Note that full information on the approval of the study protocol must also be provided in the manuscript.

Human research participants

Policy information about studies involving human research participants

Population characteristics	All participants are male or female Chinese adults. Those with one of the following criteria were excluded: serious cardiovascular diseases, thyroid dysfunction, malignant tumor, daily cigarette smoking >10 /day and daily alcohol intake >40g (male) and > 20g (female), or high-sensitive C-reactive protein level >5mg/dl.
Recruitment	All participants were recruited from those taking the elective surgery in the hospital. Written informed consents were obtained before the surgery. There is no bias when picking the participants.
Ethics oversight	This study was approved by the ethics committee of the First Affiliated Hospital of Jinan University.

Note that full information on the approval of the study protocol must also be provided in the manuscript.

Flow Cytometry

Plots

Confirm that:

- [x] The axis labels state the marker and fluorochrome used (e.g. CD4-FITC).
- [x] The axis scales are clearly visible. Include numbers along axes only for bottom left plot of group (a 'group' is an analysis of identical markers).
- [x] All plots are contour plots with outliers or pseudocolor plots.
- [x] A numerical value for number of cells or percentage (with statistics) is provided.

Methodology

Sample preparation	Adipose tissue pads were digested in 1.5mg/ml collagenase type I (Invitrogen) in DMEM for 40 min at 37℃ in CO2 incubator without shaking. The digested mixture was filtered through a 100μm cell strainer (BD Biosciences) and centrifuged at 800 x g for 10 min. The pelleted cells (SVF) were collected and washed with red blood cell lysis buffer (NH4Cl 150mM, KHCO3 10mM, Na2EDTA 0.1mM, pH=7.4) and PBS before staining with antibodies or the isotype control antibodies in PBS with 3% BSA for 1 hr at 4℃. After washing with PBS, the cells were fixed in 4% formalin.
Instrument	LSR Fortessa Analyzer (BD Biosciences)
Software	The data were analyzed with FlowJo X 10.0.7r2.

Cell population abundance	The abundance of each concerned population within single lymphocytes was provided in the figures. The single lymphocytes were gated based on FSC and SSC with the abundance of 30%~40% in all detected signals.
Gating strategy	The detailed gating strategy can be provided in the Supplementary Information if required. Briefly, the lymphocytes were gated from all cells based on SSC-A and FSC-A. Then the single cells were gated from the lymphocytes based on FSC-H and FSC-A. Within all single cells, macrophages were gated as F4/80+ and CD11b+ cells compared with the isotype controls. Then, the CD11c+ and CD206+ cells were also gated based on the corresponding isotype controls.

[x] Tick this box to confirm that a figure exemplifying the gating strategy is provided in the Supplementary Information.
